# Modeling and fitting protein-protein complexes to predict change of binding energy

**DOI:** 10.1038/srep25406

**Published:** 2016-05-13

**Authors:** Daniel F.A.R. Dourado, Samuel Coulbourn Flores

**Affiliations:** 1 Department of Cell and Molecular Biology, Computational and Systems Biology, Uppsala University, Biomedical Center Box 596, 751 24, Uppsala, Sweden

## Abstract

It is possible to accurately and economically predict change in protein-protein interaction energy upon mutation (ΔΔG), when a high-resolution structure of the complex is available. This is of growing usefulness for design of high-affinity or otherwise modified binding proteins for therapeutic, diagnostic, industrial, and basic science applications. Recently the field has begun to pursue ΔΔG prediction for homology modeled complexes, but so far this has worked mostly for cases of high sequence identity. If the interacting proteins have been crystallized in free (uncomplexed) form, in a majority of cases it is possible to find a structurally similar complex which can be used as the basis for template-based modeling. We describe how to use MMB to create such models, and then use them to predict ΔΔG, using a dataset consisting of free target structures, co-crystallized template complexes with sequence identify with respect to the targets as low as 44%, and experimental ΔΔG measurements. We obtain similar results by fitting to a low-resolution Cryo-EM density map. Results suggest that other structural constraints may lead to a similar outcome, making the method even more broadly applicable.

Modeling Protein-Protein Interactions (PPIs)^1^ is fundamentally important in biology as it probes normal as well as diseased protein function. For example, such models explain the role of Parkinson’s-disease associated mutations in Parkin^1^,^2^,^3^. PPIs are also important in the development of therapeutic and diagnostic biologics (monoclonal antibodies, or mAbs, and alternative scaffolds)^4^.

Biologics have a growing and economically substantial field of application. However raising antibodies or finding an alternative scaffold to bind a given target is difficult and time consuming. Even when starting with a scaffold that binds reasonably, affinity maturation requires a substantial experimental effort, and maintaining specificity can be a challenge^5^. Likewise protein engineering often creates many simultaneous mutations, with possible immunogenicity and solubility issues, and no insight as to which substitutions are responsible for the main effect^6^. Thus there is demand for an economical computational method which will suggest a relatively small number of substitutions which have high likelihood of improving binding.

Computational methods have made significant progress for cases where a crystallographic complex is available of the potential biologic bound to its target (we will refer to these as bound structures). Some are Molecular Dynamics (MD) based methods^7^,^8^,^9^,^10^, which typically are associated with a high computational cost. So, the applicability of such methods to large complexes or to a substantial number of mutations, which is required the case for protein-protein affinity maturation protocols, can be quite limited. On the other hand, Knowledge Based (KB) methods, which empirically combine several energetic terms including implicit solvent^11^,^12^,^13^,^14^,^15^,^16^, are fast but most perform little or no structural optimization and cannot model the backbone rearrangements induced by mutation. KB methods have also been combined with sequence analysis^17^, and interface structure alignments^18^ but this requires evolutionary information which is not available for all complexes (e.g. many biologics), and further has only been demonstrated for homology models based on high sequence identity (only 4% of their dataset had sequence identity below 50%)^17^. Recently, we described Zone Equilibration of Mutants (ZEMu)^1^, validated with 1254 mutants (1–15 simultaneous mutations) of 65 different complexes, which offers both accuracy and economy. ZEMu is implemented in MacroMoleculeBuilder (MMB)^19^,^20^, a multiscale internal-coordinate modeling code in which flexibility and an all-atom force field can be limited to regions of interest^1^,^21^. The method significantly improves the existing FoldX potential^13^, and arguably shows promise to improve others^17^,^18^ which perform limited structural minimization.

There are limited options for computing ΔΔG for the case in which the interacting proteins have only been crystallized in the free form. For many such structures, low resolution density maps of their complex are available^22^. The recent explosion in Cryo Electron Microscopy, brought about by the direct electron detector^23^, promises a rich source of new structural data, notably of complexes which are hard to crystallize. In addition, solution scattering produces many low-resolution density maps^24^, and Free-Electron Lasers promise to eventually reach comparable single-molecule resolution^25^.

Alternatively, for most structures available in the free form (referred to as targets)^26^, it is possible to find a structurally related template which can be used to build a template-based model of the complex^27^. Template modeling uses a structural alignment, which can be done accurately even at low sequence identity^27^,^28^.

This realization has led to considerable interest in template-based docking^29^. Specific cases in which the free structure is related to one of the proteins in the template complex include antibody-bound IGF-I (complex exists of the related IGF-II bound to an antibody)^30^, human Chorionic Somatomammotropin (hCS)^31^ vs. human Growth Hormone (GH) Receptor (complex exists of GH vs. GH Receptor), FcγRI vs. IgG1 (complexes have long existed of FcγRII and FcγRIII vs. IgG, while for FcγRI vs. IgG1 a mutated complex was recently solved)^32^,^33^,^34^. In this study we implement a fast and simple to use internal-coordinate template-based docking protocol in MMB^19^,^20^, that works even in the range of ~40% sequence identity for homologous proteins (quite near the twilight zone)^27^,^35^ and extend ZEMu^1^ to predict ΔΔG for thus-modeled and fitted complexes.

## Results

The template-based docking protocol introduced here results in good ΔΔG precision for homologous templates, those which (in this work) have sequence identities (vs. the targets) in the range of 44% to 51%. It is naturally more precise for self-templates, meaning those which have high sequence identity (>93%) to their targets. In this work when we provide RMSD (Root Mean Square Deviation) we refer in all cases to the discrepancy in backbone 3D atomic structure of modeled vs. template complexes. When we provide RMSE (Root Mean Square Error) and correlation, we are comparing experimental to computed ΔΔG.

Double-free models are made by docking two targets onto the template complex, while single-free models are made by docking one target onto the template (see Methods). For the double-free models based on self-templates the Root Mean Square Deviations (RMSDs) range from 0.71 to 3.42 Å (Table 1). The exception is the TGF-βType II Receptor/TGF-β3 complex for which the RMSD is 22.84 Å (Table 1). If we analyze the structures of the model and template in detail (Fig. S1) we can observe that distal region of the co-crystal chain A (TGF-β 3) is poorly resolved. In fact, if we omit template chain A residues 40 to 80 the RMSD decreases from 21.7826 Å to just 0.8819 Å, which is in line with RMSD found for the other complexes (Table 1). Since these poorly resolved residues are placed on a distal region of chain A, far from the interface, the ΔΔG prediction precision with this model is in line with that of the rest of the dataset. We describe this complex in more detail later. For the single- and double-free models based on homologous templates the RMSD ranges from 2.76 to 4.86 Å. As expected the RMSDs of this sub-group are higher than the ones observed for the double-free models based on self-templates. However the differences are relatively small, which is in line with idea that structure is more conserved than sequence^27^. We discuss this point in more detail below.

**Figure 1:**
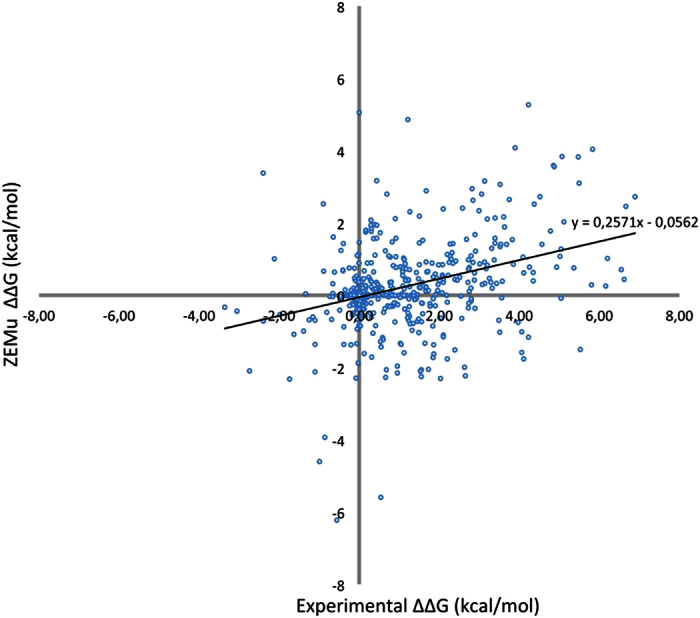
ZEMu versus experimental ΔΔG over the full dataset (846 mutants).

Based on the validation dataset described in Table 1, we compared the performance of ZEMu to FoldX-only (meaning FoldX with no prior MMB equilibration) in predicting ΔΔG. For the entire dataset the correlation between FoldX-only and experimental ΔΔG (ΔΔG_FoldX-only_ and ΔΔG_exp_, respectively) is 0.12, while the Root Mean Square Error (RMSE) is 1.83 kcal/mol. To our knowledge this is the first time FoldX is evaluated with modeled complexes; this also serves as an external (non-ZEMu) validation of our modeling protocol. For ZEMu the correlation improves to 0.34 (p-value < 0.00001); the RMSE also improves, to 1.54 kcal/mol (Table 2, Fig. 1, Tables S1 and S2).

The improvement over FoldX-only is reflected in the complete dataset as well as in the single and multiple mutant sub-groups (Table 2). For the single mutants, FoldX-only shows an RMSE of 1.89 kcal/mol and correlation of 0.06, while for ZEMu we obtain an RMSE of just 1.54 kcal/mol and a higher correlation of 0.24 (p-value = 0.00004) (Table 2, Fig. 1, Tables S1 and S2). In the case of the multiple mutants FoldX-only achieves an RMSE of 1.79 kcal/mol and correlation of 0.15, and ZEMu an RMSE of 1.54 kcal/mol and a correlation of 0.37 (p-value < 0.00001) (Table 2, Fig. 1, Tables S1 and S2).

From the main dataset we also created a sub-group comprising mutants for which self-template structures are available (Table 3). Based on this sub-group we compared the performance of ZEMu for modeled vs. crystallographic complexes. As expected, performance was better for crystallographic than for modelled complexes, but only moderately (RMSE of 1.58 vs. 1.76, Correlation of 0.61 vs. 0.38, respectively). This further highlights the quality of the modeling protocol. In the particular case of the TGF-βType II Receptor / TGF-β3 complex model, the RMSD vs. its self-template is 22.84 Å, when computed based on all resolved residues. As explained above the huge RMSD value found is due to poorly resolved residues in a distal region of chain A co-crystal (Table 1, Fig. S1) and so does not affect the quality and performance of the model at the interface. The RMSEs are 1.68 kcal/mol and 1.19 kcal/mol, and the correlations are is 0.28 and 0.83, for the model and co-crystal, respectively. The performance is thus in line with the other models.

**Figure 2:**
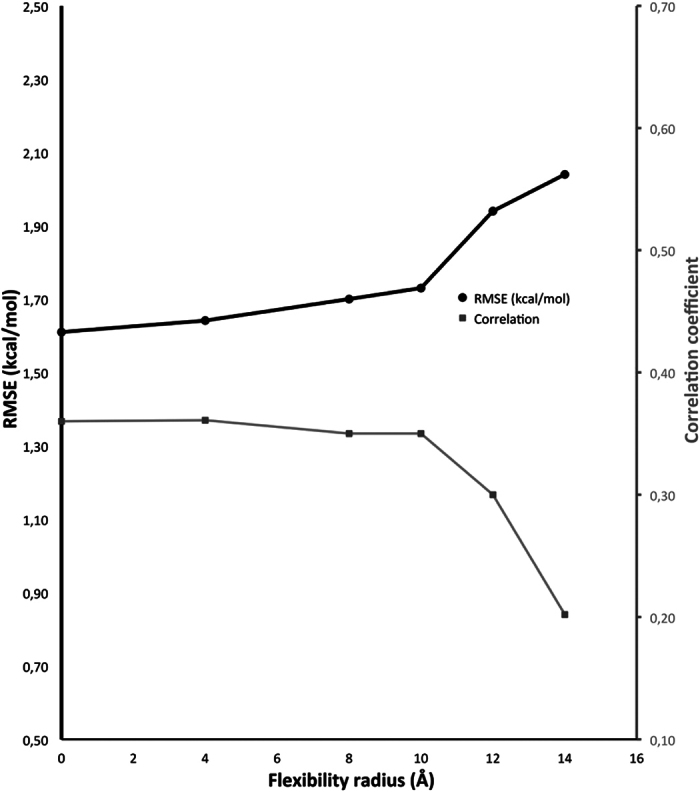
Results of flexibilizing a spatial neighbourhood of the mutation sites for a dataset composed of 687 mutants (not includes the template models of FcγRIII/IgG1 and FcγRII/IgG1 based on a homologous co-crystal complex). Ordinary ZEMu has only five flexible residues about each mutation site flexibilized. Here we also flexibilize all residues within a radius (0–14 Å) of the mutation sites. Radius of 0 Å corresponds to ordinary ZEMu.

We then tested a ZEMu variant with an additional flexibility radius (0–14 Å) (Fig. 2) centered on the mutation site, which can include residues from the binding partner. When a radius of 4 Å is used an RMSE very slightly higher than that of regular ZEMu is obtained, also an added computational cost is incurred, so there is no reason to use the added flexibility. Nonetheless, ZEMu variants with an extra flexibility radius of 4 to 10 Å still show better performance than FoldX-only.

The application to the FcγRI/IgG1 complex illustrates some of the advantages of ZEMu. We created single-free and double-free template models, and a fitted model, of this complex (Table 4, Table S2), based on an FcγRIII/IgG1 template (sequence identity of FcγRI with FcγRIII is 45%)^33^. When we performed this work no FcγRI/IgG1 co-crystal was available, but one was reported more recently^34^. With the FcγRI/IgG1 single-free template model, FoldX-only yields an RMSE of 2.33 and a correlation of 0.12 while ZEMu RMSE is just 0.92 and correlation is 0.42. The difference in performance between the two protocols is overwhelmingly due to three mutations involving N-terminus residue G236. When the three mutants are left out, FoldX-only RMSE drops substantially to 0.32 and the correlation increases to 0.32. Though this is a small sample, it illustrates even more strikingly that FoldX’s rigid backbone is particularly unsuitable for modeling the terminal region, which has characteristically high mobility.

For the double-free and fitted FcγRI/IgG1 models the three mutations involving N-terminus residue G236 are immediately adjacent to unresolved residues. The free IgG1 structure (3DNK) was missing several residues from the N-terminus (residues 229 to 235, part of the lower hinge connecting Fc to Fab in a full-length IgG1), which were resolved in the template complex (1E4K). These three mutants plus one mutation in the missing region therefore could not be modeled in the double-free and fitted models. ZEMu still outperforms FoldX-only but by a smaller margin (Table 4). Directly comparing against the double-free and fitted models, we can conclude that for both FoldX-only and ZEMu the best performance was obtained for the fitted model (Table 4). If we analyze the RMSD of both models with respect to the co-crystal structure we can observe that the fitted model has a lower RMSD by ~0.32 Å. In the fitted model the hinges between the D1 and D2 domains on FcγRI, and between the CH2 and CH3 domains on Fc, were made flexible, to allow domain motions, explaining the better RMSD of chains A and C. This highlights the efficacy of the fitting protocol.

ZEMu and MMB performance for models of FcγRIII/IgG1 and FcγRII/IgG1 based on homologous templates further demonstrates the efficacy of the protocol in cases where the self-template is not available. For instance, for the FcγRIII/IgG1 model based on its self-template the RMSE is 0.85 kcal/mol and the RMSD is 1.98 Å. In the case of the FcγRIII/IgG1 model based on the FcγRII/IgG1 template (PDB:3RY6)^32^ (sequence identity of FcγRIII with FcγRII is 51%) the RMSE increases to 1.17 kcal/mol (0.32 kcal/mol higher). The RMSD (vs. the self-template) is 4.86 Å, which is low but still 2.4 fold higher than the RMSD found for the double-free model based on a self-template. Similarly, for the FcγRII/IgG1 model based on a self-template the RMSE is 0.90 kcal/mol while the RMSD (vs. the self-template) is 2.72 Å. For the FcγRII/IgG1 model based on FcγRIII/IgG1 template (PDB:1E4K)^33^ (sequence identity of FcγRII with FcγRIII is 44%) the RMSE increases to just 1.13 kcal/mol, possibly because the RMSD increases only 1.3-fold to 3.61 Å. For these two complexes together (150 mutants, including some with single and some with multiple substitutions) the RMSE increased by 0.22 kcal/mol when lower sequence identity (44–51%) homologous templates were used in the modeling instead of high sequence identity (>93%) self-templates.

Positive Predictive Value (PPV) is defined as TP/(TP+FP) (see supporting information). In order to compute statistical quantities like this we would need a random sample of possible mutations. However many of the available experimental ΔΔG’s are the result of alanine scanning mutagenesis experiments and although 36% of the dataset have experimental ΔΔG < 0 kcal/mol, only 5% have experimental ΔΔG < −0.5 kcal/mol. Also given the interest in affinity maturation^36^ it is likely that there are more affinity-improving mutations in the peer-reviewed literature and in SKEMPI than would occur through random mutagenesis. It is thus doubtful that SKEMPI, or our dataset, contains a representative sample of substituted residue types or a representative ratio of affinity increasing vs. affinity reducing mutations. We nonetheless computed the PPV which gives the odds of obtaining ΔΔG_exp_ ≤ c_exp_ for c_exp_ = 0, −0.5 and −1.0 kcal/mol, given ΔΔG_ZEMu_ ≤ c_ZEMu_ , for several c_ZEMu_’s. Considering the entire dataset, for ΔΔG_ZEMu_ ≤ −0.5 kcal/mol, the probability of satisfying ΔΔG_exp_ ≤ −0.5 kcal/mol is 0.43. We emphasize strongly that this PPV is not applicable to the case that ΔΔG_ZEMu_ is computed for all possible point mutations at an interface, as would be done in a likely practical application.

## Discussion

In prior work^1^ we described a means to predict ΔΔG in crystallographically observed PPIs. A key goal in Structural Bioinformatics is the ability to compute ΔΔG even for the very common case of proteins whose structure is known crystallographically only in the free form. In many cases evolutionary information^17^ does not exist or is not applicable. We formed template models based on an available cocrystallized complex (which in principle could be e.g. isoforms or homologous of the free structures), with a new template modelling protocol that we describe. We here demonstrate that the protocol does not require a high sequence identity for building significant template models based on a homologous template (recall, sequence identity for homologous proteins ranged between 44% and 51%), whereas existing methods work only with high sequence identity templates^17^,^18^. For low sequence identity, our method is only moderately less precise, again related to the idea that structure is more conserved than sequence^27^. We also assembled protein complexes by flexibly fitting to a 10 Å resolution density map of an isoform protein^21^.

We emphasize that all results labelled “FoldX-only” were obtained on complexes modelled by us (albeit without subsequent ZEMu processing of the mutation sites), providing independent (non-ZEMu) validation of our template-docking protocol.

Globally, our success using template-docked and fitted models suggests that other means of docking under constraints, e.g. using biochemical or bioinformatical data, may lead to comparable results. We further suggest that if experimental ΔΔG data is available, ZEMu could be used to validate and/or refine the docked, modeled, or fitted structures. This could significantly improve docking results^37^ but is important even for template-based modeling or fitting when the constraints come from an isoform or homolog, since the binding mode may not be conserved.

Our main validation dataset consists of template modeled complexes. We used several variations of ZEMu on these complexes and evaluated the accuracy of ΔΔG prediction. More-sophisticated variants of ZEMu, which flexibilized various spatial regions, had very similar results on the main dataset when compared to the published method^1^, indicating that it is best to limit the flexibility to the immediate sequence neighborhood of the mutated residues. This validates the perturbative assumption underlying ZEMu, namely that the substitution mutations have the largest effect in the near neighborhood of the mutation site, and less effect farther from that position. Anecdotal observations suggest MD potentials introduce structural artifacts, so leaving as much as possible of the crystallographic structure unmodified may be key to ZEMu’s success. We introduce an MMB modeling protocol and show that it leads to a versatile method to predict ΔΔG on modeled and fitted protein-protein complexes.

## Methods

We built models of protein-protein complexes using MMB template modeling to align the free protein structures to relevant protein crystal complexes and also by fitting to a low-resolution density maps using ICFF^21^ (described below). We then used ZEMu^1^ to predict ΔΔG upon mutation and compared to the results of using FoldX-only^13^. Note that in all cases FoldX-only analysis was conducted with the MMB modeled or fitted structures. ZEMu was mostly used as described in^1^,^2^,^3^. Though we also tested the effect of flexibilizing additional residues in the spatial neighborhood (up to 14 Å) of the mutation sites.

### Dataset

**Figure 3:**
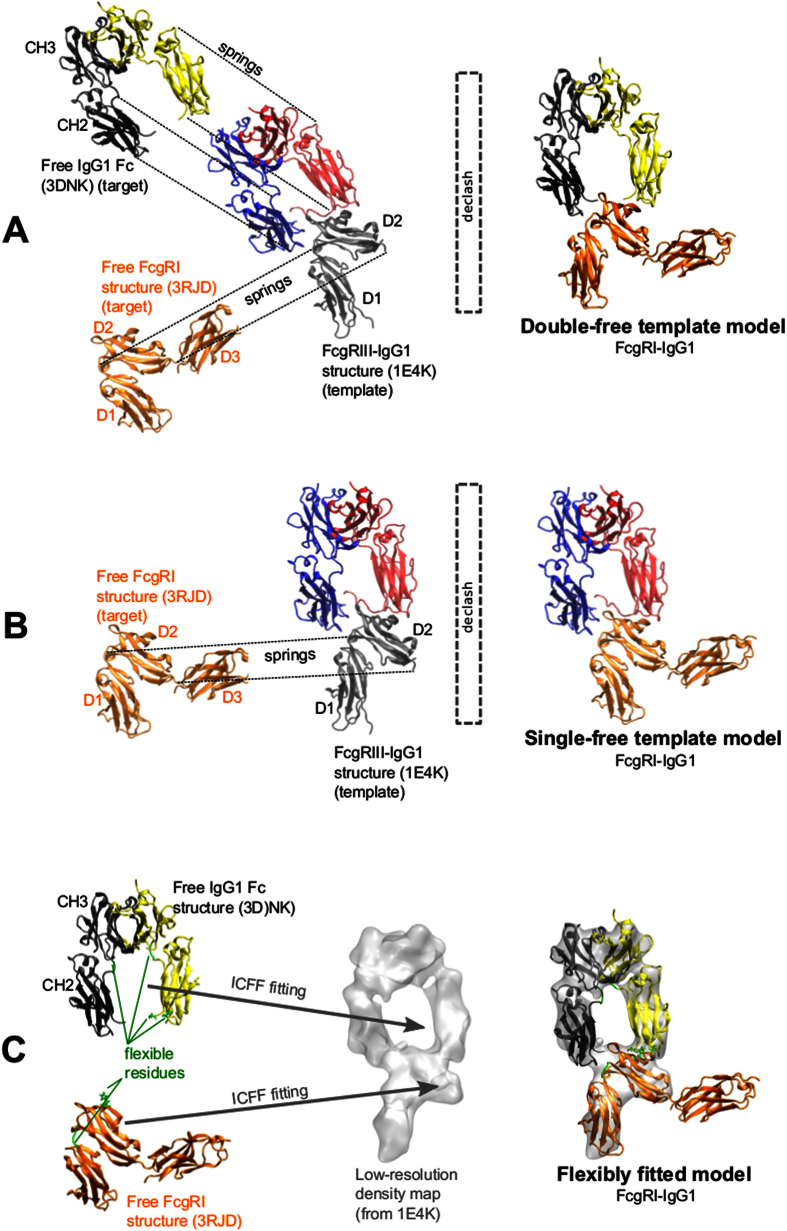
Creating complexes with MMB template modeling and ICFF. As an example, we create an FcγRI-IgG1 model based on the experimentally observed FcγRIII-IgG1 complex. (**A**) A double-free template model is created as follows. We rigidify all chains. The two chains comprising the IgG1 Fc are constrained to each other, for the free (from PDB ID: 3DNK) structure. For the template (1E4K), all chains are constrained to ground. Springs connect the binding domain (D2) of the free FcγRI (3RJD) to the D2 domain of the template FcγRIII (1E4K). The springs connect residues which correspond based on a gapped sequence alignment. Similarly, springs connect the two CH2 domains of the free to the CH2 domains of the template IgG1. Once the free are superimposed on the template proteins, both of the template proteins are deleted. The complex is then declashed (see text), leaving the modeled complex ready for ΔΔG evaluation. The models in our main dataset were prepared in this way, except for the two complexes highlighted in Table 1. (**B**) A single-free template model is prepared as above, except that we have only one target (FcγRI) and the IgG1 is retained from the template rather than being deleted. (**C**) A fitted model is created using ICFF as described in^21^. We require an electron density map, which may be at low resolution (here we created a simulated map at 10 Å resolution from 1E4K using MDFF). We rigidly and approximately fit both protein structures into the available density map. Then, we allow flexibility in hinge residues (to enable domain motions) as well as certain residues in the protein-protein interface of interest (which would otherwise clash). We activate an MD force field in the spatial neighborhood of the flexible residues. We then turn on forces which push atoms along the density gradient, until the flexible fitting has converged^21^. Note significant domain motions occurred during the fitting process. Fitting is on the basis of all domains which are present in the low-resolution density map (i.e. D3 is not fitted).

The validation dataset comprises 846 mutants (each with 1-6 simultaneous substitutions) of 11 different protein-protein complexes models for which ΔΔG_exp_ is available (Table 1)^36^,^38^,^39^,^40^,^41^,^42^,^43^,^44^,^45^,^46^,^47^,^48^,^49^,^50^,^51^,^52^,^53^,^54^,^55^,^56^,^57^,^58^,^59^,^60^. The dataset consists mostly of double-free template models (two targets). In some cases the templates were the same proteins as the targets (self-templates, Table S1); such systems are useful for benchmarking purposes. In other cases we used homologous templates (Fig. 3A; Table S2). We also created a single-free template model, for which only one of the two targets comes from a free structure, while the other is retained from the template (Fig. 3B; Table S2). Finally, we also generated a model of the biomedically important FcγRI /IgG1^61^ by fitting to a low-resolution synthetic density map of FcγRIII/IgG1^33^ using ICFF^21^.

### Template modeling in MMB

Several good template-based modeling methods exist^62^,^63^,^64^. Our procedure starts with a sequence alignment^65^ between unbound (target) and bound (template) target and template residues, followed by structural alignment. We then resolve any steric clashes, after which the model is ready to be used for ΔΔG prediction with ZEMu or potentially other purposes. The procedure (described in detail below) is straightforward and convenient to do in MMB.

### Structural alignment based on gapped sequence alignment

We have previously described morphing^21^,^66^ and homology modeling of RNA^20^ and ribonucleoprotein complexes^19^, using springs which connect residues in a rigid molecule of known structure, with corresponding residues in a flexible molecule of unknown structure. The mentioned correspondence is determined by a simple SeqAn^65^ gapped sequence alignment now available in MMB, with a mismatch and gap opening penalty of −1. In contrast with our homology modeling work (in which a fully-flexible unstructured target chain is aligned with a rigid template)^19^, or our morphing work (in which a partially-flexible structured target is aligned with a rigid template)^21^,^66^, here those springs align one or more unbound targets (or their binding domains) onto corresponding domains in the template, (Fig. 3) with both the targets and the template remaining fully rigid during the process. The template is then deleted, leaving the modeled targets in their place (Fig. 3A,B).

### Declashing

The thus-modeled complex typically has a small number of clashing residues, as the binding interface of the target was previously exposed to solvent allowing greater freedom to the side chains and to some extent the backbone. These clashes must be removed for accurate ΔΔG calculation (Fig. 3A,B). The problem of side chains that clash due to modeling is not unlike that of side chains that clash due to *in silico* mutagenesis, the problem that is addressed by ZEMu. Therefore we use a ZEMu-like method^1^ to declash. We flexibilize only the clashing residue or, if needed, a 5-residue zone centered on the main clashing residue, and equilibrate under PARM99^67^ interactions with near neighbors. We then minimize the structure under the FoldX potential^13^.

### Flexible fitting to low-resolution density maps

In addition to the template models described above, we also flexibly fitted free FcγRI and IgG1 structures into a synthetic 10 Å-resolution density map (based on PDB ID 1E4K) of the FcγRIII-IgG1 complex. We previously described Internal Coordinate Flexible Fitting (ICFF). In ICFF, each atom in the molecule (or relevant fragment thereof) perceives a force which is proportional to the electronic density gradient at the nuclear position^21^. The molecule in question can have hinge and interface flexibility, and MD forces can be applied about such points of flexibility. Hinges between the D1 and D2 domains on FcγRI, and between the CH2 and CH3 domains on Fc, were made flexible, to allow domain motions. Interface residues 1236 and 1298 (on Fc) and 148 (on FcγRI) were granted side-chain flexibility, to relieve clashes which would otherwise occur as the two subunits come into contact to form the complex (Fig. 3C).

### Evaluating ΔΔG for the modeled complexes

ZEMu involves first specifying a flexibility zone comprising five residues consecutive in sequence, centered on the mutation site. The flexibility zone is treated in torsion space, leaving the remainder of the protein rigid and fixed. Also, a physics zone of 12 Å around each flexible residue is defined, inside of which electrostatic and van der Waals forces are active. The system is then minimized by dynamics^1^.

The interaction energy of the equilibrated complex is evaluated with the Knowledge Based (KB) potential FoldX^13^. We conduct the calculation for the MMB-equilibrated wild type and mutant complexes to obtain ΔG_wild type_ and ΔG_mutant_, respectively. An estimate of ΔΔG_exp_ is obtained as follows^68^:

ΔΔG_ZEMu_ ^≡^ ΔG_mutant_ – ΔG_wild type_ ≈ ΔΔG_exp_

### ZEMu+ additional flexibility radius

We also tested a variation of ZEMu which grants flexibility to residues in the spatial neighborhood of the mutation sites. This was defined as all residues within a certain radius of the mutation site, potentially including residues of the adjacent protein. We evaluated radii ranging from 0 (equivalent to ordinary ZEMu) to 14 Å. After equilibration we evaluated ΔΔG with FoldX^13^ as before.

## Additional Information

**How to cite this article**: Dourado, D. F.A.R. and Flores, S. C. Modeling and fitting protein-protein complexes to predict change of binding energy. *Sci. Rep.* **6**, 25406; doi: 10.1038/srep25406 (2016).

## Supplementary Material

Supplementary Information

## Figures and Tables

**Table 1:** Validation dataset.

**Protein 1 (PDB)**	**Protein 2 (PDB)**	**Complex**	**MODEL**	**Sequence identity**	**Model vs template RMSD (Å)**	**Number of mutants (subtitutions)**
IgG1-K D44.1 FAB (1MLB)	Hen egg-white lysozyme (1DPX)	1MLC	IgG1-K D44.1 FAB/Hen egg-white lysozyme	1MLC:A,B/1MLB:A=100% 1MLC:E/1DPX:A=100%	global = 0.98 chain A = 0.96 chain B = 1.10 chain E = 0.75	16(26)
IgG1-K D1.3 FV (1VFA)	Hen egg-white lysozyme (1DPX)	1VFB	IGG1-KAPPA D1.3 FV/Hen egg-white lysozyme	1VFB:A/1VFA:A=100% 1VFB:C/1DPX:A=100%	global = 0.80 chain A = 0.33 chain B = 0.68 chain C = 1.19	42(56)
HyHEL-63 FAB (1DQM)	Hen egg-white lysozyme (1DPX)	1DQJ	HyHEL-63 FAB/Hen egg-white lysozyme	1DQJ:A/1DQM:A=100% 1DQJ:C/1DPX:A=100%	global = 0.80 chain A = 0.69 chain B = 0.91 chain C = 0.78	34(47)
TGF-β Type II Receptor (1M9Z)	TGF-β 3 (1TGJ)	1KTZ	TGF-β Type II Receptor/TGF-β 3	1KTZ:A/1TGJ:A=100% 1KTZ:A/1M9Z:A=98.2%	global = 22.84 global (without 40–80) = 0.88 chain A = 21.79 chain A(without 40–80) = 1.21 chain B = 0.63	27(27)
β-lactamase inhibitor protein-I (3GMU)	TEM-1 β-lactamase (1ZG4)	1JTG	β-lactamase inhibitor protein-I/TEM-1 β-lactamase	1JTG:A/:3GMU:A=100% 1JTG:B/1ZG4:A=98.2%	global = 3.42 chain A = 1.94 chain B = 0.64	143(307)
IgG1 (4DZ8)	Fragment B of protein A (2JWD)	1FC2	IgG1/Fragment B of protein A	1FC2:C/2JWD:A=93.1% 1FC2:D/4DZ8:A=96.4%	global = 1.67 chain C = 0.27 chain D = 1.35	9(9)
Iso-1-cytochrome C (1NMI)	Cytochrome C peroxidase (3R99)	2PCC	Iso-1-Cytochrome C/Cytochrome C peroxidase	2PCC:A/3R99:A=99.3% 2PCC:B/1NMI:A=99.1%	global = 1.21 chain A = 0.40 chain B = 2.18	12(18)
IgG1 (3DNK)	FcγR II (3RY4)	3RY6	IgG1/FcγR II	3RY6:C/3RY4:A=97.1% 3RY6:A/3DNK:A=98.8%	global = 2.72 chain A = 1.90 chain B = 3.62 chain C = 2.22	65(138)
IgG1 (3DNK)	FcγR III (1E4J)	1E4K	IgG1/FcγR III	1E4K:C/1E4J:A =100% 1E4K:A/3DNK:A=97.2%	global = 1.99 chain A = 1.59 chain B = 2.55 chain C = 1.59	95(155)
IgG1 (3DNK)	FcγR N (4N0F)	4N0U	IgG1/FcγR N	4N0U:A/4N0F:A=100% 4N0U:A/3DNK:A=97.1%	global = 0.71 chain A = 0.31 chain E = 1.01	53(53)
IgG1 (1E4K)	FcγR I (3RJD)	1E4K(IgG1/FcγR III)	IgG1/FcγR I	1E4K:C/3RJD:A=45.2%	global = 2.76 chain A = 2.80 chain B = 2.34 chain C = 2.80	66(146)
IgG1 (3DNK)	FcγR II (3RY4)	1E4K(IgG1/FcγR III)	Ig1/FcγR II	1E4K:C/3RY4:A=44.0% 1E4K:A/3DNK:A=97.2%	global = 3.61 chain A = 3.10 chain B = 4.23 chain C = 3.36	65(138)
IgG1 (3DNK)	FcγR III (1E4J)	3RY6(IgG1/FcγR II)	IgG1/FcγR III	3RY6:C/1E4J:A=50.9% 3RY6:A/3DNK:A=98.8%	global = 4.86 chain A = 4.79 chain B = 4.61 chain C = 4.88	95(155)
IgG1 (3DNK)	FcγR I (3RJD)	1E4K(IgG1/FcγR III)	IgG1/FcγR I	1E4K:C/3RJD:A=45.2% 1E4K:A/3DNK:A=97.2%	global = 3.68 chain A = 3.03 chain B = 2.52 chain C = 4.80	62(128)
IgG1 (3DNK)	FcγR I (3RJD)	Density map from 1E4K	IgG1/FcγR I	1E4K:C/3RJD:A=45.2% 1E4K:A/3DNK:A=97.2%	global = 3.37 chain A = 2.71 chain B = 2.56 chain C = 4.30	62(128)
					TOTAL	846(1531)

The dataset is divided in two groups. The first is composed of double-free template models, based on self-templates. The second group (bottom of table, separated by a blank row) includes : 1) a single-free template model of IgG1/Fcγ R I, based on IgG1/Fcγ R III crystal, where the structure of IgG1 from the crystallographic complex is kept rather than being replaced; 2) double-free template models of IgG1/Fcγ R I, IgG1/Fcγ R II and IgG1/Fcγ R III, which were modeled from based on homologous templates; 3) A model of IgG1/FcγR I built by fitting to a low-resolution density map synthesized from an IgG1/FcγR III crystallographic complex^21^ Sequence identity and backbone RMSD of targets vs. templates are shown.

**Table 2:** Comparison between Foldx-only and ZEMu performance.

Dataset	Number of mutants	Models			
		**FoldX-only**		**ZEMu**	
		**RMSE (kcal/mol)**	**Correlation**	**RMSE (kcal/mol)**	**Correlation**
All mutants	846	1.83	0.12	1.54	0.34
Multiple mutants	584	1.79	0.15	1.54	0.37
Single mutants	262	1.89	0.06	1.54	0.24

**Table 3:** Comparing the performance of ZEMu on modeled vs. crystallographic complexes.

Number of mutants	Double-free Models				Co-crystals	
	FoldX-only		ZEMu		ZEMu	
	RMSE (kcal/mol)	Correlation	RMSE (kcal/mol)	Correlation	RMSE (kcal/mol)	Correlation
558	2.00	0.17	1.76	0.38	1.58	0.61

Note that ZEMu performance decreased only moderately (1.76 vs. 1.58 RMSE) for modeled vs. crystallographic complexes.

**Table 4:** Comparison between Foldx-only and ZEMu performance for FcγRI/IgG1 single-free and double-free template-based and fitted models.

MODEL	Number of mutants	FoldX-only		ZEMu	
		RMSE (kcal/mol)	Correlation	RMSE (kcal/mol)	Correlation
Single-free template-based model	66	2.33	0.12	0.92	0.42
Double-free template-based model	62	0.64	0.41	0.57	0.49
Fitted model	62	0.49	0.47	0.47	0.50
