# Supplementary material for: Modeling and fitting protein-protein complexes to predict change of binding energy
Source: Sci Rep. 2016 May 13;6:25406. doi: 10.1038/srep25406 (PMC4865953; doi:10.1038/srep25406)
Supplement: Supplementary Information [file srep25406-s1.doc]

## Modeling and fitting protein-protein complexes to predict change of binding energy

Daniel F.A.R. Dourado. Samuel Coulbourn Flores*

[Department of Cell and Molecular Biology. *Computational and Systems Biology*](http://katalog.uu.se/orgInfo/?orgId=X62:11), Uppsala university, Biomedical Center Box 596, 751 24, Uppsala, Sweden

*Correspondence and requests for materials should be addressed to S.F. (email: [sam@xray.bmc.uu.se](mailto:sam@xray.bmc.uu.se))

**Supporting information**

### Computing Positive Predictive Value (PPV)

How confident are we that the predicted ΔΔG is “low/high enough” by an experimental criterion given a computational criterion? The more stringent a computational criterion is, the higher the confidence that the experimental criterion will be met. The experimental criterion can be defined as:

ΔΔG_exp_ ≤ c_exp_

The *computational criterion* is:

ΔΔG_ZEMu_ ≤ c_ZEMu_

Where

c _ZEMu_ ≤ 0,

c_exp_ ≤ 0

We define a True Positive (TP) as the event that :

ΔΔG_ZEMu_ ≤ c_ZEMu_ AND ΔΔG_exp_ ≤ c_exp_

Similarly, we define a False Positive (FP) as the event that:

ΔΔG_ZEMu_ ≤ c_ZEMu_ AND ΔΔG_exp_ > c_exp_

The Positive Predictive Value (PPV):

PPV = TP / (TP+FP)

### Definitions

Equation 1 - Root Mean Square Error (RMSE)

$$\text{RMSE=}\sqrt{\frac{1}{\text{N}}\sum_{\text{i=1}}^{\text{N}} \text{(}\Delta\Delta\text{G}_{\text{i,computed}}-\Delta\Delta\text{G}_{\text{i,experimental}}\text{)}^{2}}$$

Equation 2 - FoldX energy function ^18^

$$\begin{matrix} \Delta G=\Delta G_{Electrostatic}+W_{VDW}\bullet\Delta G_{VDW}+\Delta G_{hbond}+\Delta G_{waterbridges}+W_{solventapolar}\bullet\Delta G_{solvenapolar} \\ +W_{solventPolar}\bullet\Delta G_{solventPolar}+W_{backbone}\bullet T\Delta S_{backbone}+W_{sidechain}\bullet T\Delta S_{sidechain}+\Delta G_{Str}+\Delta G_{Kon} \end{matrix}$$

*∆G_Electrostatic_* = electrostatic contribution; *∆G_vdw_* = van der Waals contribution; *∆G_Hbond_* = difference in the free energy between an intra-molecular hydrogen bond and an inter-molecular hydrogen bond; *∆G_Waterbridges_* = free energy difference associated with a water molecule establishing more than one hydrogen bond with protein residues; *∆G_solventPolar_* = free energy difference of the polar groups solvent energy in the protein unfolded and folded state. *∆G_solventApolar_* = free energy difference of the apolar groups solvent energy in the protein unfolded and folded state; *T∆S_backbone_* = entropic energy associated with having the backbone fixed; *T∆S_sidechain_* = entropic energy associated with having the sidechain fixed; *∆S_tr_* = entropic cost associated with the formation of the protein-protein complex; *∆S_kon_* = electrostatic effect on the association constant; All *W* terms are weight factors.


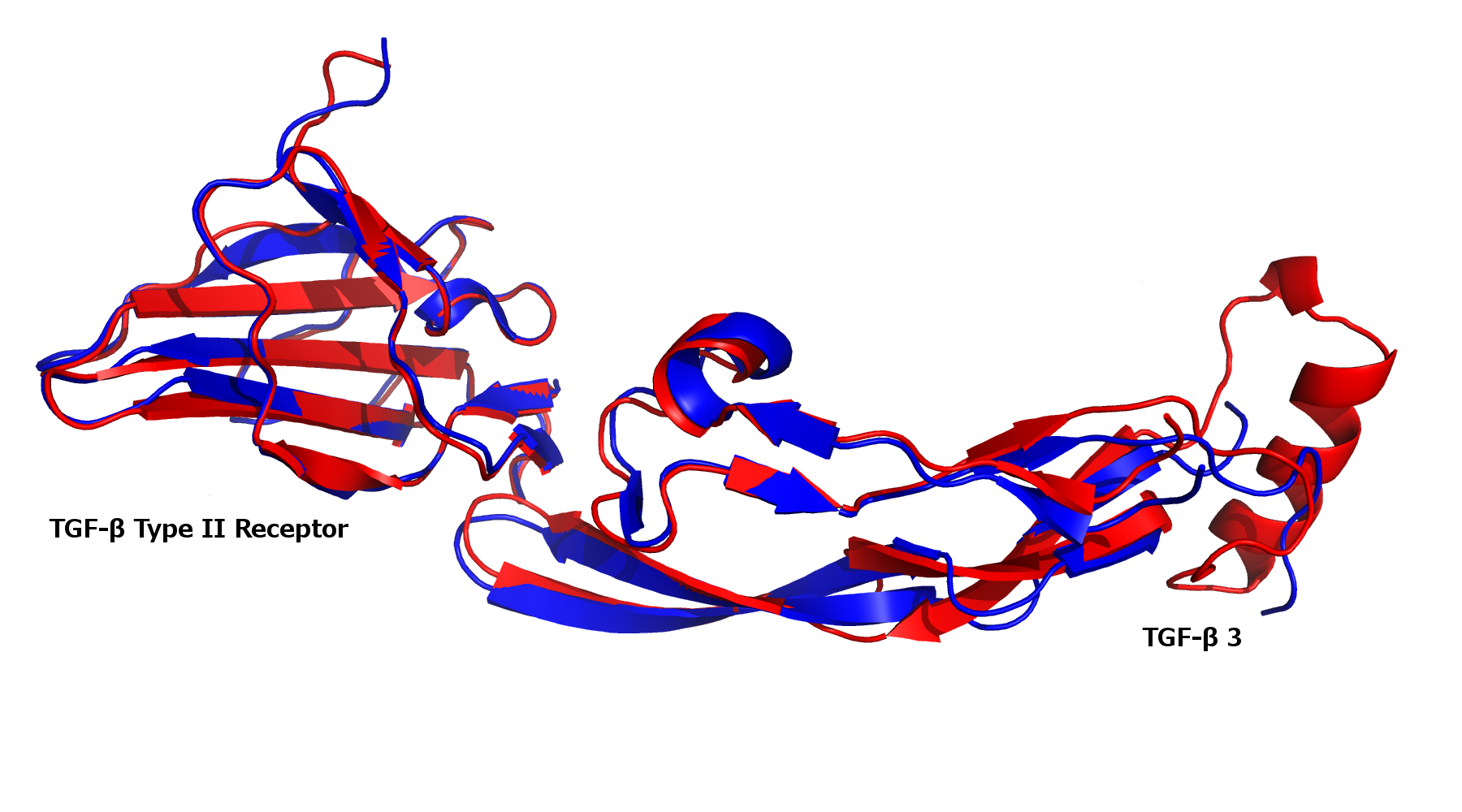


Figure S1. TGF-β Type II Receptor/TGF-β 3 complex. Model (Red) vs. co-crystal 1KTZ (Blue).

Table S1

Sub-group 1 of the validation dataset. Composed of double-free models, based on self- (effectively identical, rather than homologous) templates (see text). ZEMu vs. experimental ΔΔG. Mutant nomenclature follows the pattern: **CWNNS**, where **C** is the chain ID, **W** is the wild type residue type, **NN** (or **NNN**) is the residue number, and **S** is the substituted (mutant) residue type.

| **MODEL** | **Mutant** | **ΔΔGexp (kcal/mol)** | **number of substitutions** | **ΔΔG ZEMu (kcal/mol)** |
| --- | --- | --- | --- | --- |
|  |  |  |  |  |
| IGG1:ΚD44.1 FAB /HEN EGG WHITE LYSOZYME | AN32G | -0.86 | 1 | -3.93 |
| IGG1:ΚD44.1 FAB /HEN EGG WHITE LYSOZYME | AN32Y | 0.00 | 1 | -0.09 |
| IGG1:ΚD44.1 FAB /HEN EGG WHITE LYSOZYME | BS57V | -0.49 | 1 | -0.41 |
| IGG1:ΚD44.1 FAB /HEN EGG WHITE LYSOZYME | BS57A | -0.37 | 1 | 0.02 |
| IGG1:ΚD44.1 FAB /HEN EGG WHITE LYSOZYME | BT58D | -0.56 | 1 | -6.22 |
| IGG1:ΚD44.1 FAB /HEN EGG WHITE LYSOZYME | AN92A | -1.25 | 1 | -0.68 |
| IGG1:ΚD44.1 FAB /HEN EGG WHITE LYSOZYME | BT31V | 0.53 | 1 | 0.22 |
| IGG1:ΚD44.1 FAB /HEN EGG WHITE LYSOZYME | BT31A | 0.45 | 1 | 0.13 |
| IGG1:ΚD44.1 FAB /HEN EGG WHITE LYSOZYME | BT31W | 0.13 | 1 | -0.57 |
| IGG1:ΚD44.1 FAB /HEN EGG WHITE LYSOZYME | BT28D | -0.15 | 1 | 0.01 |
| IGG1:ΚD44.1 FAB /HEN EGG WHITE LYSOZYME | BK65D | 0.02 | 1 | -0.22 |
| IGG1:ΚD44.1 FAB /HEN EGG WHITE LYSOZYME | BS57V. BT58D | -0.99 | 2 | -4.60 |
| IGG1:ΚD44.1 FAB /HEN EGG WHITE LYSOZYME | AN32G. AN92A | -0.69 | 2 | -0.67 |
| IGG1:ΚD44.1 FAB /HEN EGG WHITE LYSOZYME | AN32G. AN92A. AS57V. AT58D | -1.74 | 4 | -2.32 |
| IGG1:ΚD44.1 FAB /HEN EGG WHITE LYSOZYME | AN92A. BS57V. BT58D | -2.74 | 4 | -2.09 |
| IGG1:ΚD44.1 FAB /HEN EGG WHITE LYSOZYME | AN92A. B28T. BS57V. BT58D | -2.40 | 3 | 3.38 |
| β-lactamase inhibitor protein-I/TEM-1 β-lactamase | B163K | -1.98 | 1 | -0.53 |
| β-lactamase inhibitor protein-I/TEM-1 beta-lactamase | B163K. B135K. B134K | -3.05 | 3 | -0.44 |
| β-lactamase inhibitor protein-I/TEM-1 β-lactamase | B165K. B163K. B135K. B89K | -3.36 | 4 | -0.33 |
| β-lactamase inhibitor protein-I/TEM-1 β-lactamase | B165K. B163K. B89K | -2.39 | 3 | -0.71 |
| β-lactamase inhibitor protein-I/TEM-1 β-lactamase | B32K | 0.20 | 1 | -0.06 |
| β-lactamase inhibitor protein-I/TEM-1 beta-lactamase | B140K. B157K | -0.40 | 2 | 1.43 |
| β-lactamase inhibitor protein-I/TEM-1 β-lactamase | B74A | 3.56 | 1 | -0.07 |
| β-lactamase inhibitor protein-I/TEM-1 β-lactamase | B74A. A104A | 1.58 | 2 | -0.22 |
| β-lactamase inhibitor protein-I/TEM-1 β-lactamase | B74A. A104A. A105A | 1.39 | 3 | -0.12 |
| β-lactamase inhibitor protein-I/TEM-1 β-lactamase | KB74A. YA105A | 3.32 | 2 | 0.20 |
| β-lactamase inhibitor protein-I/TEM-1 β-lactamase | B74A. B142A. B143A. A130A | 5.06 | 4 | 3.84 |
| β-lactamase inhibitor protein-I/TEM-1 β-lactamase | B74A. A234A | 6.55 | 2 | 0.71 |
| β-lactamase inhibitor protein-I/TEM-1 β-lactamase | B74A. B142A. A234A | 5.83 | 3 | 4.04 |
| β-lactamase inhibitor protein-I/TEM-1 β-lactamase | B74A. B142A. B143A. A234A | 5.47 | 4 | 3.83 |
| β-lactamase inhibitor protein-I/TEM-1 β-lactamase | B74A. B143A. A234A | 4.87 | 3 | 3.56 |
| β-lactamase inhibitor protein-I/TEM-1 β-lactamase | B74A. A243A | 6.62 | 2 | 0.44 |
| β-lactamase inhibitor protein-I/TEM-1 β-lactamase | B74A. B142A | 4.85 | 2 | 3.59 |
| β-lactamase inhibitor protein-I/TEM-1 β-lactamase | B74A. B142A. A104A | 3.13 | 3 | 3.16 |
| β-lactamase inhibitor protein-I/TEM-1 β-lactamase | B74A. B142A. A104A. A105A | 3.13 | 4 | 0.92 |
| β-lactamase inhibitor protein-I/TEM-1 β-lactamase | B74A. B142A. A105A | 4.78 | 2 | 1.78 |
| β-lactamase inhibitor protein-I/TEM-1 β-lactamase | B74A. B142A. A243A | 5.49 | 2 | 3.10 |
| β-lactamase inhibitor protein-I/TEM-1 β-lactamase | B74A. B142A. B143A | 3.89 | 2 | 4.08 |
| β-lactamase inhibitor protein-I/TEM-1 β-lactamase | B74A. B142A. B143A. A104A | 2.39 | 4 | 0.44 |
| β-lactamase inhibitor protein-I/TEM-1 β-lactamase | B74A. B142A. B143A. A104A. A105A | 2.41 | 5 | 0.95 |
| β-lactamase inhibitor protein-I/TEM-1 β-lactamase | B74A. B142A. B143A. A105A | 4.23 | 4 | 5.27 |
| β-lactamase inhibitor protein-I/TEM-1 β-lactamase | B74A. B143A | 3.06 | 2 | 2.80 |
| β-lactamase inhibitor protein-I/TEM-1 β-lactamase | B74A. B143A. A104A | 1.05 | 3 | -1.36 |
| β-lactamase inhibitor protein-I/TEM-1 β-lactamase | B74A. B143A. A104A. A105A | 1.77 | 4 | -0.23 |
| β-lactamase inhibitor protein-I/TEM-1 β-lactamase | B74A. B143A. A105A | 3.39 | 3 | 2.14 |
| β-lactamase inhibitor protein-I/TEM-1 β-lactamase | B74A. B143A. A243A | 4.37 | 3 | 2.52 |
| β-lactamase inhibitor protein-I/TEM-1 β-lactamase | B89K | -0.45 | 1 | -0.04 |
| β-lactamase inhibitor protein-I/TEM-1 β-lactamase | B93K | -0.48 | 1 | -0.03 |
| β-lactamase inhibitor protein-I/TEM-1 β-lactamase | A243A. A234A | 2.73 | 2 | 0.72 |
| β-lactamase inhibitor protein-I/TEM-1 β-lactamase | A243A. A235A. A234A | 2.72 | 3 | 0.36 |
| β-lactamase inhibitor protein-I/TEM-1 β-lactamase | NA100A | -0.46 | 1 | 1.24 |
| β-lactamase inhibitor protein-I/TEM-1 β-lactamase | NA100A. WB112A | 2.79 | 2 | -0.16 |
| β-lactamase inhibitor protein-I/TEM-1 β-lactamase | NA100A. FB142A | 2.29 | 2 | 1 |
| β-lactamase inhibitor protein-I/TEM-1 β-lactamase | NA100A. HB148A | 2.10 | 2 | -1.10 |
| β-lactamase inhibitor protein-I/TEM-1 β-lactamase | NA100A. WB150A | 4.35 | 2 | 0.40 |
| β-lactamase inhibitor protein-I/TEM-1 β-lactamase | NA100A. RB160A | 1.58 | 2 | 0.35 |
| β-lactamase inhibitor protein-I/TEM-1 β-lactamase | NA100A. WB162A | 2.10 | 2 | -1.15 |
| β-lactamase inhibitor protein-I/TEM-1 β-lactamase | NA100A. KB74A | 3.51 | 2 | -1.02 |
| β-lactamase inhibitor protein-I/TEM-1 β-lactamase | VA103A | 1.91 | 1 | 0.02 |
| β-lactamase inhibitor protein-I/TEM-1 β-lactamase | VA103A. FB142A | 4.51 | 2 | 2.73 |
| β-lactamase inhibitor protein-I/TEM-1 β-lactamase | VA103A. RB160A | 4.35 | 2 | 0.63 |
| β-lactamase inhibitor protein-I/TEM-1 β-lactamase | VA103A. WB162A | 4.23 | 2 | -1.16 |
| β-lactamase inhibitor protein-I/TEM-1 β-lactamase | EA104A | 1.55 | 1 | -2.25 |
| β-lactamase inhibitor protein-I/TEM-1 β-lactamase | EA104A. SB113A | 1.86 | 2 | -1.62 |
| β-lactamase inhibitor protein-I/TEM-1 β-lactamase | EA110A. SB113A. SB71A | 5.04 | 3 | 1.28 |
| β-lactamase inhibitor protein-I/TEM-1 β-lactamase | EA104K | 4.23 | 1 | not converged |
| β-lactamase inhibitor protein-I/TEM-1 β-lactamase | PA107A | -0.38 | 1 | -1.36 |
| β-lactamase inhibitor protein-I/TEM-1 β-lactamase | PA107A. HB41A | 2.65 | 2 | -2.23 |
| β-lactamase inhibitor protein-I/TEM-1 β-lactamase | PA107A. YB53A | 2.39 | 2 | -1.51 |
| β-lactamase inhibitor protein-I/TEM-1 β-lactamase | EA110A | 4.06 | 1 | -1.58 |
| β-lactamase inhibitor protein-I/TEM-1 β-lactamase | EA110A. SB113A | 4.56 | 2 | 1.41 |
| β-lactamase inhibitor protein-I/TEM-1 β-lactamase | EA110A. SB113A. SB71A | 5.04 | 3 | -0.08 |
| β-lactamase inhibitor protein-I/TEM-1 β-lactamase | EA110A. SB71A | 5.02 | 2 | 1.08 |
| β-lactamase inhibitor protein-I/TEM-1 β-lactamase | MA129A | 0.74 | 1 | 1.09 |
| β-lactamase inhibitor protein-I/TEM-1 β-lactamase | MA129A. SB113A. SB71A | 1.67 | 3 | 2.89 |
| β-lactamase inhibitor protein-I/TEM-1 β-lactamase | MA129A. FB36A | 3.63 | 2 | 1.89 |
| β-lactamase inhibitor protein-I/TEM-1 β-lactamase | MA129A. YB53A | 3.61 | 2 | 2.17 |
| β-lactamase inhibitor protein-I/TEM-1 β-lactamase | EA168A | -0.07 | 1 | -0.14 |
| β-lactamase inhibitor protein-I/TEM-1 β-lactamase | EA168A. WB112A | 2.79 | 2 | 1.09 |
| β-lactamase inhibitor protein-I/TEM-1 β-lactamase | EA168A. FB142A | 2.58 | 2 | 2.41 |
| β-lactamase inhibitor protein-I/TEM-1 β-lactamase | EA168A. WB150A | 4.11 | 2 | 0.62 |
| β-lactamase inhibitor protein-I/TEM-1 β-lactamase | EA168A. RB160A | 2.32 | 2 | 1.18 |
| β-lactamase inhibitor protein-I/TEM-1 β-lactamase | EA168A. WB162A | 2.08 | 2 | 0.51 |
| β-lactamase inhibitor protein-I/TEM-1 β-lactamase | EA168A. KB74A | 4.06 | 2 | -1.01 |
| β-lactamase inhibitor protein-I/TEM-1 β-lactamase | VA216A | -0.41 | 1 | 0.68 |
| β-lactamase inhibitor protein-I/TEM-1 β-lactamase | SA235A. SA130A. KA234A | 1.85 | 3 | 0.51 |
| β-lactamase inhibitor protein-I/TEM-1 β-lactamase | RA243A. SA235A. SA130A | 1.69 | 3 | 0.65 |
| β-lactamase inhibitor protein-I/TEM-1 β-lactamase | QA99A | 0.43 | 1 | 0.36 |
| β-lactamase inhibitor protein-I/TEM-1 β-lactamase | QA99A. WB112A | 3.54 | 2 | 1.41 |
| β-lactamase inhibitor protein-I/TEM-1 β-lactamase | QA99A. FB142A | 2.82 | 2 | 2.95 |
| β-lactamase inhibitor protein-I/TEM-1 β-lactamase | QA99A. HB148A | 3.20 | 2 | 0.83 |
| β-lactamase inhibitor protein-I/TEM-1 β-lactamase | QA99A. WB150A | 3.82 | 2 | 1.54 |
| β-lactamase inhibitor protein-I/TEM-1 β-lactamase | QA99A. RB160A | 3.75 | 2 | 2.65 |
| β-lactamase inhibitor protein-I/TEM-1 β-lactamase | QA99A. WB162A | 2.89 | 2 | 1.68 |
| β-lactamase inhibitor protein-I/TEM-1 β-lactamase | QA99A. KB74A | 4.11 | 2 | -1.76 |
| β-lactamase inhibitor protein-I/TEM-1 β-lactamase | WB112A | 3.01 | 1 | 0.87 |
| β-lactamase inhibitor protein-I/TEM-1 β-lactamase | SB113A | -0.17 | 1 | -0.21 |
| β-lactamase inhibitor protein-I/TEM-1 β-lactamase | FB142A. EA104A | 2.75 | 2 | 0.77 |
| β-lactamase inhibitor protein-I/TEM-1 β-lactamase | FB142A. YB143A. EA104A. YA105A | 2.84 | 4 | 0.91 |
| β-lactamase inhibitor protein-I/TEM-1 β-lactamase | HB148A | 2.75 | 1 | 0.11 |
| β-lactamase inhibitor protein-I/TEM-1 β-lactamase | WB150A | 4.25 | 1 | 0.78 |
| β-lactamase inhibitor protein-I/TEM-1 β-lactamase | RB160A | 2.22 | 1 | 0.55 |
| β-lactamase inhibitor protein-I/TEM-1 β-lactamase | WB162A | 2.34 | 1 | 1.43 |
| β-lactamase inhibitor protein-I/TEM-1 β-lactamase | FB36A | 3.20 | 1 | 2.11 |
| β-lactamase inhibitor protein-I/TEM-1 β-lactamase | HB41A | 3.25 | 1 | 0.47 |
| β-lactamase inhibitor protein-I/TEM-1 β-lactamase | YB53A | 2.08 | 1 | 0.56 |
| β-lactamase inhibitor protein-I/TEM-1 β-lactamase | SB71A | 0.36 | 1 | 1.57 |
| β-lactamase inhibitor protein-I/TEM-1 β-lactamase | A238S | -1.63 | 1 | -1.08 |
| β-lactamase inhibitor protein-I/TEM-1 β-lactamase | A104A. A105A | 1.03 | 2 | -1.33 |
| β-lactamase inhibitor protein-I/TEM-1 β-lactamase | A104K. B142A | 4.55 | 2 | not converged |
| β-lactamase inhibitor protein-I/TEM-1 β-lactamase | A105A | -0.17 | 1 | -0.50 |
| β-lactamase inhibitor protein-I/TEM-1 β-lactamase | A130A | 0.79 | 1 | 0.26 |
| β-lactamase inhibitor protein-I/TEM-1 β-lactamase | A234A | 1.03 | 1 | 0.79 |
| β-lactamase inhibitor protein-I/TEM-1 β-lactamase | A234A. A130A | 1.98 | 2 | 0.84 |
| β-lactamase inhibitor protein-I/TEM-1 β-lactamase | A234A. A130A. A243A | 2.51 | 3 | 0.50 |
| β-lactamase inhibitor protein-I/TEM-1 β-lactamase | A234A. A235A. A130A. A243A | 2.79 | 4 | 0.20 |
| β-lactamase inhibitor protein-I/TEM-1 β-lactamase | A235A | 1.24 | 1 | -0.10 |
| β-lactamase inhibitor protein-I/TEM-1 β-lactamase | A238S. B142A | 3.68 | 2 | 1.41 |
| β-lactamase inhibitor protein-I/TEM-1 β-lactamase | A243A | 1.27 | 1 | -0.79 |
| β-lactamase inhibitor protein-I/TEM-1 β-lactamase | A243A. A130A | 1.85 | 2 | -0.09 |
| β-lactamase inhibitor protein-I/TEM-1 β-lactamase | B140K | -0.01 | 1 | 1.15 |
| β-lactamase inhibitor protein-I/TEM-1 β-lactamase | B142A | 2.10 | 1 | 2.39 |
| β-lactamase inhibitor protein-I/TEM-1 β-lactamase | B142A. A104A. A105A | 1.50 | 3 | 0.22 |
| β-lactamase inhibitor protein-I/TEM-1 β-lactamase | B142A. A105A | 0.69 | 2 | 2.80 |
| β-lactamase inhibitor protein-I/TEM-1 β-lactamase | B142A. A243A | 3.39 | 2 | 1.58 |
| β-lactamase inhibitor protein-I/TEM-1 β-lactamase | B142A. B143A | 2.84 | 2 | 2.63 |
| β-lactamase inhibitor protein-I/TEM-1 β-lactamase | B142A. B143A. A104A | 2.89 | 3 | -0.36 |
| β-lactamase inhibitor protein-I/TEM-1 β-lactamase | B142A. B143A. A105A | 2.99 | 3 | 2.34 |
| β-lactamase inhibitor protein-I/TEM-1 β-lactamase | B143A | 0.38 | 1 | 1.96 |
| β-lactamase inhibitor protein-I/TEM-1 β-lactamase | B143A. A104A | 1.86 | 2 | -1.37 |
| β-lactamase inhibitor protein-I/TEM-1 β-lactamase | B143A. A104A. A105A | 2.05 | 3 | -0.63 |
| β-lactamase inhibitor protein-I/TEM-1 β-lactamase | B143A. A105A | 1.07 | 2 | 0.04 |
| β-lactamase inhibitor protein-I/TEM-1 β-lactamase | B143A. A243A | 2.27 | 2 | 1.08 |
| β-lactamase inhibitor protein-I/TEM-1 β-lactamase | B163A | -1.34 | 1 | 0.04 |
| β-lactamase inhibitor protein-I/TEM-1 β-lactamase | B-49-A | 2.56 | 1 | -0.64 |
| β-lactamase inhibitor protein-I/TEM-1 β-lactamase | A-104-K.B-49-A | 5.64 | 2 |  |
| β-lactamase inhibitor protein-I/TEM-1 β-lactamase | A-110-A.B-50-A | 1.50 | 2 | 0.85 |
| β-lactamase inhibitor protein-I/TEM-1 β-lactamase | A-168-A.B-49-A | 3.97 | 2 | -0.75 |
| β-lactamase inhibitor protein-I/TEM-1 β-lactamase | A-238-S.B-49-A | 2.63 | 2 | -1.99 |
| β-lactamase inhibitor protein-I/TEM-1 β-lactamase | A-234-A.B-49-A | 1.55 | 2 | -2.06 |
| β-lactamase inhibitor protein-I/TEM-1 β-lactamase | A-234-A.A-130-A.B-49-A | 1.32 | 3 | -0.97 |
| β-lactamase inhibitor protein-I/TEM-1 β-lactamase | A-234-A.A-130-A.A-243-A.B-49-A | 1.49 | 4 | -1.03 |
| β-lactamase inhibitor protein-I/TEM-1 β-lactamase | A-234-A.A-235-A.A-130-A.A-243-A.B-49-A | 1.73 | 5 | -2.05 |
| β-lactamase inhibitor protein-I/TEM-1 β-lactamase | A-129-A.B-50-A | 0.36 | 2 | 0.65 |
| β-lactamase inhibitor protein-I/TEM-1 β-lactamase | A-107-A.B-50-A | -0.65 | 2 | 1.61 |
| β-lactamase inhibitor protein-I/TEM-1 β-lactamase | A-243-A.B-49-A | 1.11 | 2 | 1.03 |
| β-lactamase inhibitor protein-I/TEM-1 β-lactamase | A-243-A.A-234-A.B-49-A | 1.90 | 3 | 0.59 |
| β-lactamase inhibitor protein-I/TEM-1 β-lactamase | A-243-A.A-130-A.B-49-A | 1.25 | 3 | -0.31 |
| β-lactamase inhibitor protein-I/TEM-1 β-lactamase | A-243-A.A-235-A.B-49-A | 0.41 | 3 | -0.84 |
| β-lactamase inhibitor protein-I/TEM-1 β-lactamase | A-243-A.A-235-A.A-234-A.B-49-A | 2.03 | 4 | -2.30 |
| β-lactamase inhibitor protein-I/TEM-1 β-lactamase | A-243-A.A-235-A.A-130-A.B-49-A | 0.89 | 4 | -0.74 |
| β-lactamase inhibitor protein-I/TEM-1 β-lactamase | A-130-A.B-49-A | 1.38 | 2 | -0.07 |
| β-lactamase inhibitor protein-I/TEM-1 β-lactamase | A-235-A.B-49-A | 1.64 | 2 | -1.33 |
| β-lactamase inhibitor protein-I/TEM-1 β-lactamase | A-235-A.A-234-A.B-49-A | 1.53 | 3 | -2.10 |
| β-lactamase inhibitor protein-I/TEM-1 β-lactamase | A-235-A.A-130-A.B-49-A | 1.09 | 3 | 0.26 |
| β-lactamase inhibitor protein-I/TEM-1 β-lactamase | A-235-A.A-130-A.A-234-A.B-49-A | 0.61 | 4 | -0.47 |
| β-lactamase inhibitor protein-I/TEM-1 β-lactamase | B-50-A | -0.41 | 1 | 0.70 |
| β-lactamase inhibitor protein-I/TEM-1 β-lactamase | A-99-A.B-49-A | 2.20 | 2 | -0.19 |
| β-lactamase inhibitor protein-I/TEM-1 β-lactamase | A-216-A.B-50-A | -0.89 | 2 | 0.67 |
| IGG1:ΚD1.3 FV/HEN EGG WHITE LYSOZYME | HA30A | 0.84 | 1 | -0.30 |
| IGG1:ΚD1.3 FV/HEN EGG WHITE LYSOZYME | YA32A | 1.72 | 1 | 0.66 |
| IGG1:ΚD1.3 FV/HEN EGG WHITE LYSOZYME | YA49A | 0.80 | 1 | -0.29 |
| IGG1:ΚD1.3 FV/HEN EGG WHITE LYSOZYME | YA50A | 0.52 | 1 | 1.73 |
| IGG1:ΚD1.3 FV/HEN EGG WHITE LYSOZYME | WA92A | 2.73 | 1 | 0.98 |
| IGG1:ΚD1.3 FV/HEN EGG WHITE LYSOZYME | SA93A | 0.34 | 1 | -1.05 |
| IGG1:ΚD1.3 FV/HEN EGG WHITE LYSOZYME | TB30A | -0.06 | 1 | 0.00 |
| IGG1:ΚD1.3 FV/HEN EGG WHITE LYSOZYME | YB32A | 0.46 | 1 | 0.31 |
| IGG1:ΚD1.3 FV/HEN EGG WHITE LYSOZYME | WB52A | 0.92 | 1 | 1.07 |
| IGG1:ΚD1.3 FV/HEN EGG WHITE LYSOZYME | DB54A | 0.64 | 1 | -0.37 |
| IGG1:ΚD1.3 FV/HEN EGG WHITE LYSOZYME | NB56A | 0.18 | 1 | -0.02 |
| IGG1:ΚD1.3 FV/HEN EGG WHITE LYSOZYME | DB58A | -0.21 | 1 | -1.37 |
| IGG1:ΚD1.3 FV/HEN EGG WHITE LYSOZYME | EB98A | 1.16 | 1 | 0.46 |
| IGG1:ΚD1.3 FV/HEN EGG WHITE LYSOZYME | RB99A | -0.10 | 1 | -0.12 |
| IGG1:ΚD1.3 FV/HEN EGG WHITE LYSOZYME | DB100A | 3.07 | 1 | 0.30 |
| IGG1:ΚD1.3 FV/HEN EGG WHITE LYSOZYME | YB101F | 0.95 | 1 | -1.96 |
| IGG1:ΚD1.3 FV/HEN EGG WHITE LYSOZYME | DC18A | 0.34 | 1 | 0.18 |
| IGG1:ΚD1.3 FV/HEN EGG WHITE LYSOZYME | NC19A | 0.40 | 1 | 1.11 |
| IGG1:ΚD1.3 FV/HEN EGG WHITE LYSOZYME | YC23A | 0.41 | 1 | 0.18 |
| IGG1:ΚD1.3 FV/HEN EGG WHITE LYSOZYME | SC24A | 0.85 | 1 | 0.82 |
| IGG1:ΚD1.3 FV/HEN EGG WHITE LYSOZYME | KC116A | 0.71 | 1 | -0.29 |
| IGG1:ΚD1.3 FV/HEN EGG WHITE LYSOZYME | TC118A | 0.77 | 1 | 0.36 |
| IGG1:ΚD1.3 FV/HEN EGG WHITE LYSOZYME | DC119A | 0.95 | 1 | -2.15 |
| IGG1:ΚD1.3 FV/HEN EGG WHITE LYSOZYME | VC120A | 0.92 | 1 | 0.77 |
| IGG1:ΚD1.3 FV/HEN EGG WHITE LYSOZYME | QC121A | 2.88 | 1 | 0.42 |
| IGG1:ΚD1.3 FV/HEN EGG WHITE LYSOZYME | IC124A | 1.23 | 1 | 0.43 |
| IGG1:ΚD1.3 FV/HEN EGG WHITE LYSOZYME | RC125A | 1.84 | 1 | -0.85 |
| IGG1:ΚD1.3 FV/HEN EGG WHITE LYSOZYME | LC129A | 0.17 | 1 | -0.23 |
| IGG1:ΚD1.3 FV/HEN EGG WHITE LYSOZYME | YA32A. QC121A | 1.59 | 2 | 1.52 |
| IGG1:ΚD1.3 FV/HEN EGG WHITE LYSOZYME | YA32A. IC124A | 2.91 | 2 | 1.22 |
| IGG1:ΚD1.3 FV/HEN EGG WHITE LYSOZYME | YA50A. DC18A | 1.27 | 2 | 2.31 |
| IGG1:ΚD1.3 FV/HEN EGG WHITE LYSOZYME | YA50A. DC119A | 1.23 | 2 | -0.69 |
| IGG1:ΚD1.3 FV/HEN EGG WHITE LYSOZYME | WA92A. QC121A | 3.52 | 2 | 3.07 |
| IGG1:ΚD1.3 FV/HEN EGG WHITE LYSOZYME | WA92A. IC124A | 3.85 | 2 | 0.86 |
| IGG1:ΚD1.3 FV/HEN EGG WHITE LYSOZYME | WA92A. RC125A | 3.39 | 2 | 1.44 |
| IGG1:ΚD1.3 FV/HEN EGG WHITE LYSOZYME | WA92A. LC129A | 3.31 | 2 | 1.30 |
| IGG1:ΚD1.3 FV/HEN EGG WHITE LYSOZYME | YB32A. KC116A | 1.59 | 2 | -0.51 |
| IGG1:ΚD1.3 FV/HEN EGG WHITE LYSOZYME | WB52A. DC119A | 2.25 | 2 | 0.91 |
| IGG1:ΚD1.3 FV/HEN EGG WHITE LYSOZYME | DB54A. TC118A | 1.25 | 2 | -0.58 |
| IGG1:ΚD1.3 FV/HEN EGG WHITE LYSOZYME | DB100A. SC24A | 3.37 | 2 | 0.98 |
| IGG1:ΚD1.3 FV/HEN EGG WHITE LYSOZYME | YB101F. DC119A | 2.74 | 2 | -0.22 |
| IGG1:ΚD1.3 FV/HEN EGG WHITE LYSOZYME | YB101F. VC120A | 2.54 | 2 | -0.02 |
| TGF-β Type II Receptor/TGF-β 3 | VA92I | 0.24 | 1 | 0.50 |
| TGF-β Type II Receptor/TGF-β 3 | RA25K | 1.15 | 1 | 0.87 |
| TGF-β Type II Receptor/TGF-β 3 | RA94K | 2.20 | 1 | -1.76 |
| TGF-β Type II Receptor/TGF-β 3 | RA25A | 1.48 | 1 | 0.46 |
| TGF-β Type II Receptor/TGF-β 3 | RA94A | 2.88 | 1 | 0.62 |
| TGF-β Type II Receptor/TGF-β 3 | LB27A | 2.27 | 1 | 1.42 |
| TGF-β Type II Receptor/TGF-β 3 | FB30A | 3.43 | 1 | 0.33 |
| TGF-β Type II Receptor/TGF-β 3 | DB32A | 1.97 | 1 | 0.30 |
| TGF-β Type II Receptor/TGF-β 3 | DB32N | 2.45 | 1 | 0.42 |
| TGF-β Type II Receptor/TGF-β 3 | SB49A | 0.77 | 1 | 0.01 |
| TGF-β Type II Receptor/TGF-β 3 | IB50A | 2.34 | 1 | 0.89 |
| TGF-β Type II Receptor/TGF-β 3 | TB51A | 1.96 | 1 | 0.13 |
| TGF-β Type II Receptor/TGF-β 3 | SB52A | 0.66 | 1 | -2.27 |
| TGF-β Type II Receptor/TGF-β 3 | SB52L | 4.48 | 1 | not converged |
| TGF-β Type II Receptor/TGF-β 3 | IB53A | 1.82 | 1 | 0.89 |
| TGF-β Type II Receptor/TGF-β 3 | EB55A | 1.66 | 1 | 0.12 |
| TGF-β Type II Receptor/TGF-β 3 | VB77A | 0.86 | 1 | 0.01 |
| TGF-β Type II Receptor/TGF-β 3 | DB118A | 1.26 | 1 | 0.24 |
| TGF-β Type II Receptor/TGF-β 3 | EB119A | 1.94 | 1 | 0.47 |
| TGF-β Type II Receptor/TGF-β 3 | EB119Q | 2.07 | 1 | 0.07 |
| TGF-β Type II Receptor/TGF-β 3 | NB47A | 0.73 | 1 | 0.00 |
| TGF-β Type II Receptor/TGF-β 3 | VB62A | 1.09 | 1 | 0.00 |
| TGF-β Type II Receptor/TGF-β 3 | EB75A | 1.53 | 1 | 0.21 |
| TGF-β Type II Receptor/TGF-β 3 | HB79A | 0.74 | 1 | -0.04 |
| TGF-β Type II Receptor/TGF-β 3 | FB110A | 1.38 | 1 | 0.00 |
| TGF-β Type II Receptor/TGF-β 3 | MB112A | 1.32 | 1 | 0.00 |
| TGF-β Type II Receptor/TGF-β 3 | IB125A | 0.99 | 1 | 0.00 |
| IMMUNOGLOBULIN FC/FRAGMENT B OF PROTEIN A COMPLEX | YC133W | 0.82 | 1 | 1.48 |
| IMMUNOGLOBULIN FC/FRAGMENT B OF PROTEIN A COMPLEX | IC135W | 0.58 | 1 | -1.17 |
| IMMUNOGLOBULIN FC/FRAGMENT B OF PROTEIN A COMPLEX | FC149W | -0.04 | 1 | -0.01 |
| IMMUNOGLOBULIN FC/FRAGMENT B OF PROTEIN A COMPLEX | LC163W | 2.18 | 1 | 0.00 |
| IMMUNOGLOBULIN FC/FRAGMENT B OF PROTEIN A COMPLEX | LC136D | 1.23 | 1 | 0.41 |
| IMMUNOGLOBULIN FC/FRAGMENT B OF PROTEIN A COMPLEX | NC147A | 0.41 | 1 | 0.14 |
| IMMUNOGLOBULIN FC/FRAGMENT B OF PROTEIN A COMPLEX | FC149A | 3.14 | 1 | 0.33 |
| IMMUNOGLOBULIN FC/FRAGMENT B OF PROTEIN A COMPLEX | IC150A | 0.00 | 1 | 0.11 |
| IMMUNOGLOBULIN FC/FRAGMENT B OF PROTEIN A COMPLEX | KC154A | 0.00 | 1 | 1.47 |
| ISO-1-CYTOCHROME C/CYTOCHROME C PEROXIDASE | DA34A | -0.90 | 1 | 2.53 |
| ISO-1-CYTOCHROME C/CYTOCHROME C PEROXIDASE | VA197A | 2.10 | 1 | 0.44 |
| ISO-1-CYTOCHROME C/CYTOCHROME C PEROXIDASE | EA290A | 6.20 | 1 | 1.02 |
| ISO-1-CYTOCHROME C/CYTOCHROME C PEROXIDASE | KB87A | 0.90 | 1 | 2.12 |
| ISO-1-CYTOCHROME C/CYTOCHROME C PEROXIDASE | KB87A. DA34A | 0.20 | 2 | 0.89 |
| ISO-1-CYTOCHROME C/CYTOCHROME C PEROXIDASE | KB87A. VA197A | 1.50 | 2 | 2.19 |
| ISO-1-CYTOCHROME C/CYTOCHROME C PEROXIDASE | KB72A | 0.30 | 1 | -0.61 |
| ISO-1-CYTOCHROME C/CYTOCHROME C PEROXIDASE | KB72A. VA197A | 2.80 | 2 | -0.10 |
| ISO-1-CYTOCHROME C/CYTOCHROME C PEROXIDASE | KB72A. EA290A | 1.10 | 2 | -0.01 |
| ISO-1-CYTOCHROME C/CYTOCHROME C PEROXIDASE | AB81G | 1.90 | 1 | -0.29 |
| ISO-1-CYTOCHROME C/CYTOCHROME C PEROXIDASE | AB81G. DA34A | -0.10 | 2 | 0.41 |
| ISO-1-CYTOCHROME C/CYTOCHROME C PEROXIDASE | AB81G. VA197A | 2.10 | 2 | 0.36 |
| HYHEL-63 FAB/HEN EGG WHITE LYSOZYME | YC20A | 3.29 | 1 | 0.36 |
| HYHEL-63 FAB/HEN EGG WHITE LYSOZYME | RC21A | 1.17 | 1 | 0.73 |
| HYHEL-63 FAB/HEN EGG WHITE LYSOZYME | WC63A | 1.35 | 1 | -0.78 |
| HYHEL-63 FAB/HEN EGG WHITE LYSOZYME | KC97A | 3.52 | 1 | 1.35 |
| HYHEL-63 FAB/HEN EGG WHITE LYSOZYME | DC101A | 1.45 | 1 | -0.72 |
| HYHEL-63 FAB/HEN EGG WHITE LYSOZYME | WC62A | 0.76 | 1 | 0.01 |
| HYHEL-63 FAB/HEN EGG WHITE LYSOZYME | LC75A | 1.45 | 1 | 0.02 |
| HYHEL-63 FAB/HEN EGG WHITE LYSOZYME | TC89A | 0.84 | 1 | 0.19 |
| HYHEL-63 FAB/HEN EGG WHITE LYSOZYME | NC93A | 0.65 | 1 | 1.23 |
| HYHEL-63 FAB/HEN EGG WHITE LYSOZYME | KC96A | 6.16 | 1 | 0.26 |
| HYHEL-63 FAB/HEN EGG WHITE LYSOZYME | SC100A | 0.78 | 1 | 1.06 |
| HYHEL-63 FAB/HEN EGG WHITE LYSOZYME | NA31A | 2.01 | 1 | 0.41 |
| HYHEL-63 FAB/HEN EGG WHITE LYSOZYME | NA32A | 4.09 | 1 | 1.06 |
| HYHEL-63 FAB/HEN EGG WHITE LYSOZYME | YA50A | 2.68 | 1 | 1.55 |
| HYHEL-63 FAB/HEN EGG WHITE LYSOZYME | SA91A | 1.43 | 1 | -0.09 |
| HYHEL-63 FAB/HEN EGG WHITE LYSOZYME | YA96A | 1.14 | 1 | -0.38 |
| HYHEL-63 FAB/HEN EGG WHITE LYSOZYME | DB32A | 2.01 | 1 | -0.62 |
| HYHEL-63 FAB/HEN EGG WHITE LYSOZYME | YB33A | 5.52 | 1 | -1.50 |
| HYHEL-63 FAB/HEN EGG WHITE LYSOZYME | YB50A | 6.89 | 1 | 2.73 |
| HYHEL-63 FAB/HEN EGG WHITE LYSOZYME | YB53A | 1.18 | 1 | 0.00 |
| HYHEL-63 FAB/HEN EGG WHITE LYSOZYME | WB98A | 4.93 | 1 | 0.78 |
| HYHEL-63 FAB/HEN EGG WHITE LYSOZYME | NA32A. KC96A | 5.80 | 2 | 0.29 |
| HYHEL-63 FAB/HEN EGG WHITE LYSOZYME | SA91A. RC21A | 3.17 | 2 | 0.74 |
| HYHEL-63 FAB/HEN EGG WHITE LYSOZYME | SA91A. YC20A | 3.57 | 2 | 0.39 |
| HYHEL-63 FAB/HEN EGG WHITE LYSOZYME | YA96A. RC21A | 3.49 | 2 | -0.69 |
| HYHEL-63 FAB/HEN EGG WHITE LYSOZYME | YA96A. SC100A | 0.91 | 2 | -0.22 |
| HYHEL-63 FAB/HEN EGG WHITE LYSOZYME | DB32A. KC97A | 1.12 | 2 | -1.59 |
| HYHEL-63 FAB/HEN EGG WHITE LYSOZYME | YB53A. WC62A | 1.29 | 2 | 0.00 |
| HYHEL-63 FAB/HEN EGG WHITE LYSOZYME | YB53A. WC63A | 2.20 | 2 | 1.13 |
| HYHEL-63 FAB/HEN EGG WHITE LYSOZYME | YB53A. LC75A | 1.22 | 2 | -0.49 |
| HYHEL-63 FAB/HEN EGG WHITE LYSOZYME | YB53A. DC101A | 2.73 | 2 | -0.35 |
| HYHEL-63 FAB/HEN EGG WHITE LYSOZYME | WB98A. SC100A | 5.36 | 2 | 0.76 |
| HYHEL-63 FAB/HEN EGG WHITE LYSOZYME | WB98A. KC97A | 6.66 | 2 | 2.46 |
| HYHEL-63 FAB/HEN EGG WHITE LYSOZYME | WB98A. YC20A | 5.11 | 2 | 2.03 |
| IGG1/FcγR II | PA238A.PB238A | 0.57 | 1 | 0.19 |
| IGG1/FcγR II | DA265A.DB265A | 1.57 | 1 | Not converged |
| IGG1/FcγR II | NA297A.NB297A | 1.77 | 1 | -0.30 |
| IGG1/FcγR II | AA327Q.AB327Q | 1.21 | 1 | -0.62 |
| IGG1/FcγR II | PA329A.PB329A | 1.50 | 1 | 1.45 |
| IGG1/FcγR II | DA270A.DB270A | 1.67 | 1 | 0.20 |
| IGG1/FcγR II | QA295A.QB295A | 0.28 | 1 | -0.50 |
| IGG1/FcγR II | AA327S.AB327S | 0.87 | 1 | -0.21 |
| IGG1/FcγR II | TA256A.TB256A | -0.20 | 1 | 0.00 |
| IGG1/FcγR II | KA290A.KB290A | -0.16 | 1 | 0.02 |
| IGG1/FcγR II | RA255A.RB255A | -0.16 | 1 | 0.00 |
| IGG1/FcγR II | EA258A.EB258A | -0.17 | 1 | 0.01 |
| IGG1/FcγR II | SA267A.SB267A | -0.25 | 1 | 0.76 |
| IGG1/FcγR II | EA272A.EB272A | -0.12 | 1 | -0.04 |
| IGG1/FcγR II | NA276A.NB276A | -0.15 | 1 | 0.01 |
| IGG1/FcγR II | DA280A.DB280A | -0.17 | 1 | 0.00 |
| IGG1/FcγR II | HA285A.HB285A | -0.14 | 1 | 0.00 |
| IGG1/FcγR II | NA286A.NB286A | -0.13 | 1 | 0.00 |
| IGG1/FcγR II | TA307A.TB307A | -0.04 | 1 | 0.00 |
| IGG1/FcγR II | LA309A.LB309A | -0.07 | 1 | 0.00 |
| IGG1/FcγR II | NA315A.NB315A | -0.08 | 1 | 0.00 |
| IGG1/FcγR II | KA326A.KB326A | -0.12 | 1 | 0.48 |
| IGG1/FcγR II | PA331A.PB331A | -0.15 | 1 | -0.26 |
| IGG1/FcγR II | SA337A.SB337A | -0.12 | 1 | 0.00 |
| IGG1/FcγR II | AA378Q.AB378Q | -0.20 | 1 | 0.00 |
| IGG1/FcγR II | EA430A.EB430A | -0.13 | 1 | 0.00 |
| IGG1/FcγR II | HA268A.HB268A | -0.11 | 1 | Not converged |
| IGG1/FcγR II | RA301A.RB301A | -0.08 | 1 | -2.29 |
| IGG1/FcγR II | KA322A.KB322A | -0.09 | 1 | 0.01 |
| IGG1/FcγR II | RA292A.RB292A | 0.78 | 1 | 0.05 |
| IGG1/FcγR II | KA414A.KB414A | 0.26 | 1 | 0.00 |
| IGG1/FcγR II | SA298A.SB298A | 0.54 | 1 | -5.59 |
| IGG1/FcγR II | SA239A.SB239A | 0.19 | 1 | 0.04 |
| IGG1/FcγR II | EA269A.EB269A | 0.26 | 1 | Not converged |
| IGG1/FcγR II | EA293A.EB293A | -0.05 | 1 | 0.00 |
| IGG1/FcγR II | YA296F.YB296F | 0.02 | 1 | 0.02 |
| IGG1/FcγR II | VA303A.VB303A | 0.09 | 1 | -0.02 |
| IGG1/FcγR II | AA327G.AB327G | 0.05 | 1 | 0.02 |
| IGG1/FcγR II | KA338A.KB338A | 0.15 | 1 | 0.00 |
| IGG1/FcγR II | DA376A.DB376A | 0.13 | 1 | 0.00 |
| IGG1/FcγR II | EA333A.EB333A | 0.05 | 1 | 0.01 |
| IGG1/FcγR II | KA334A.KB334A | -0.01 | 1 | 0.04 |
| IGG1/FcγR II | AA339T.AB339T | -0.05 | 1 | 0.00 |
| IGG1/FcγR II | IA253A.IB253A | -0.08 | 1 | 0.00 |
| IGG1/FcγR II | SA254A.SB254A | 0.02 | 1 | 0.00 |
| IGG1/FcγR II | KA288A.KB288A | -0.08 | 1 | 0.00 |
| IGG1/FcγR II | VA305A.VB305A | -0.07 | 1 | 0.00 |
| IGG1/FcγR II | QA311A.QB311A | -0.06 | 1 | 0.01 |
| IGG1/FcγR II | DA312A.DB312A | -0.11 | 1 | 0.00 |
| IGG1/FcγR II | KA317A.KB317A | -0.07 | 1 | 0.00 |
| IGG1/FcγR II | KA360A.KB360A | -0.07 | 1 | 0.53 |
| IGG1/FcγR II | QA362A.QB362A | -0.02 | 1 | 0.00 |
| IGG1/FcγR II | EA380A.EB380A | -0.10 | 1 | 0.00 |
| IGG1/FcγR II | EA382A.EB382A | 0.03 | 1 | 0.00 |
| IGG1/FcγR II | SA415A.SB415A | 0.06 | 1 | 0.00 |
| IGG1/FcγR II | SA424A.SB424A | -0.02 | 1 | 0.00 |
| IGG1/FcγR II | HA433A.HB433A | 0.05 | 1 | 0.00 |
| IGG1/FcγR II | NA434A.NB434A | 0.02 | 1 | 0.00 |
| IGG1/FcγR II | HA435A.HB435A | 0.15 | 1 | 0.00 |
| IGG1/FcγR II | YA436A.YB436A | 0.04 | 1 | 0.00 |
| IGG1/FcγR II | GA236A.GB236A | -1.16 | 1 | -0.60 |
| IGG1/FcγR II | IA332E.IB332E | -0.15 | 1 | -0.49 |
| IGG1/FcγR II | IA332E.IB332E.GA236A.GB236A | -1.39 | 2 | -0.99 |
| IGG1/FcγR II | SA239D.SB239D.IA332E.IB332E | -0.81 | 2 | -0.47 |
| IGG1/FcγR II | SA239D.SB239D.IA332E.GA236A.IB332E.GB236A | -2.12 | 3 | 1.01 |
| IGG1/FcγR III | PA238A.PB238A | 1.57 | 1 | Not converged |
| IGG1/FcγR III | DA265A.DB265A | 1.43 | 1 | 0.63 |
| IGG1/FcγR III | NA297A.NB297A | 2.08 | 1 | 0.85 |
| IGG1/FcγR III | AA327Q.AB327Q | 1.67 | 1 | 0.23 |
| IGG1/FcγR III | PA329A.PB329A | 0.92 | 1 | 1.58 |
| IGG1/FcγR III | DA270A.DB270A | 1.16 | 1 | 0.41 |
| IGG1/FcγR III | QA295A.QB295A | 0.82 | 1 | 0.00 |
| IGG1/FcγR III | AA327S.AB327S | 1.67 | 1 | 0.92 |
| IGG1/FcγR III | TA256A.TB256A | -0.16 | 1 | 0.00 |
| IGG1/FcγR III | KA290A.KB290A | -0.16 | 1 | 0.01 |
| IGG1/FcγR III | RA255A.RB255A | 0.01 | 1 | 0.00 |
| IGG1/FcγR III | EA258A.EB258A | -0.07 | 1 | 0.01 |
| IGG1/FcγR III | SA267A.SB267A | -0.03 | 1 | -1.87 |
| IGG1/FcγR III | EA272A.EB272A | 0.13 | 1 | -0.10 |
| IGG1/FcγR III | NA276A.NB276A | 0.03 | 1 | -0.08 |
| IGG1/FcγR III | DA280A.DB280A | -0.05 | 1 | 0.00 |
| IGG1/FcγR III | HA285A.HB285A | 0.08 | 1 | 0.00 |
| IGG1/FcγR III | NA286A.NB286A | -0.03 | 1 | 0.00 |
| IGG1/FcγR III | TA307A.TB307A | -0.05 | 1 | 0.10 |
| IGG1/FcγR III | LA309A.LB309A | -0.04 | 1 | 0.00 |
| IGG1/FcγR III | NA315A.NB315A | -0.04 | 1 | 0.00 |
| IGG1/FcγR III | KA326A.KB326A | -0.12 | 1 | 0.75 |
| IGG1/FcγR III | PA331A.PB331A | -0.05 | 1 | 0.06 |
| IGG1/FcγR III | SA337A.SB337A | 0.04 | 1 | 0.00 |
| IGG1/FcγR III | AA378Q.AB378Q | -0.10 | 1 | 0.00 |
| IGG1/FcγR III | EA430A.EB430A | -0.11 | 1 | 0.00 |
| IGG1/FcγR III | HA268A.HB268A | 0.36 | 1 | -0.49 |
| IGG1/FcγR III | RA301A.RB301A | 0.90 | 1 | -0.01 |
| IGG1/FcγR III | KA322A.KB322A | 0.28 | 1 | 0.00 |
| IGG1/FcγR III | RA292A.RB292A | 0.07 | 1 | 0.03 |
| IGG1/FcγR III | KA414A.KB414A | 0.12 | 1 | 0.00 |
| IGG1/FcγR III | SA298A.SB298A | -0.17 | 1 | -0.55 |
| IGG1/FcγR III | SA239A.SB239A | 0.80 | 1 | 0.37 |
| IGG1/FcγR III | EA269A.EB269A | 0.47 | 1 | -0.10 |
| IGG1/FcγR III | EA293A.EB293A | 0.69 | 1 | -0.01 |
| IGG1/FcγR III | YA296F.YB296F | 0.35 | 1 | 0.01 |
| IGG1/FcγR III | VA303A.VB303A | 0.66 | 1 | 0.01 |
| IGG1/FcγR III | AA327G.AB327G | 0.60 | 1 | -1.22 |
| IGG1/FcγR III | KA338A.KB338A | 1.12 | 1 | 0.00 |
| IGG1/FcγR III | DA376A.DB376A | 0.35 | 1 | 0.00 |
| IGG1/FcγR III | EA333A.EB333A | -0.14 | 1 | 0.20 |
| IGG1/FcγR III | KA334A.KB334A | -0.20 | 1 | -0.01 |
| IGG1/FcγR III | AA339T.AB339T | -0.17 | 1 | 0.00 |
| IGG1/FcγR III | IA253A.IB253A | -0.05 | 1 | 0.00 |
| IGG1/FcγR III | SA254A.SB254A | 0.19 | 1 | 0.00 |
| IGG1/FcγR III | KA288A.KB288A | -0.03 | 1 | 0.01 |
| IGG1/FcγR III | VA305A.VB305A | 0.10 | 1 | 0.01 |
| IGG1/FcγR III | QA311A.QB311A | 0.04 | 1 | 0.00 |
| IGG1/FcγR III | DA312A.DB312A | -0.12 | 1 | 0.00 |
| IGG1/FcγR III | KA317A.KB317A | -0.06 | 1 | 0.00 |
| IGG1/FcγR III | KA360A.KB360A | -0.12 | 1 | 0.00 |
| IGG1/FcγR III | QA362A.QB362A | -0.02 | 1 | 0.31 |
| IGG1/FcγR III | EA380A.EB380A | 0.05 | 1 | 0.00 |
| IGG1/FcγR III | EA382A.EB382A | 0.16 | 1 | 0.00 |
| IGG1/FcγR III | SA415A.SB415A | 0.09 | 1 | 0.00 |
| IGG1/FcγR III | SA424A.SB424A | 0.08 | 1 | 0.00 |
| IGG1/FcγR III | HA433A.HB433A | -0.01 | 1 | 0.00 |
| IGG1/FcγR III | NA434A.NB434A | 0.15 | 1 | 0.00 |
| IGG1/FcγR III | HA435A.HB435A | 0.15 | 1 | 0.00 |
| IGG1/FcγR III | YA436A.YB436A | 0.06 | 1 | 0.00 |
| IGG1/FcγR III | LC124Y | 0.68 | 1 | -2.06 |
| IGG1/FcγR III | QC125K | 0.43 | 1 | 0.08 |
| IGG1/FcγR III | NC126D | -0.03 | 1 | 0.03 |
| IGG1/FcγR III | FC153Y | 0.16 | 1 | 0.24 |
| IGG1/FcγR III | LC157K | -0.03 | 1 | -0.29 |
| IGG1/FcγR III | SC160Q | 0.11 | 1 | 0.46 |
| IGG1/FcγR III | KC161L | 0.55 | 1 | -1.43 |
| IGG1/FcγR III | NC162D | 0.12 | 1 | -0.19 |
| IGG1/FcγR III | SC164E | 0.15 | 1 | 0.18 |
| IGG1/FcγR III | EC36H | -0.10 | 1 | 0.00 |
| IGG1/FcγR III | DC37A | -0.31 | 1 | 0.00 |
| IGG1/FcγR III | SC66A | -0.20 | 1 | 0.00 |
| IGG1/FcγR III | SC79A | -0.11 | 1 | 0.01 |
| IGG1/FcγR III | DC80A | -0.29 | 1 | 0.00 |
| IGG1/FcγR III | KC101A | -0.03 | 1 | 0.00 |
| IGG1/FcγR III | EC102A | 0.19 | 1 | 0.00 |
| IGG1/FcγR III | EC103A | -0.07 | 1 | 0.00 |
| IGG1/FcγR III | DC104A | 0.60 | 1 | 0.00 |
| IGG1/FcγR III | SC112A | 0.04 | 1 | 0.01 |
| IGG1/FcγR III | WC113F | 1.73 | 1 | 0.13 |
| IGG1/FcγR III | QC125A | 1.36 | 1 | 0.08 |
| IGG1/FcγR III | NC126A | 1.56 | 1 | -0.21 |
| IGG1/FcγR III | CC127A | 0.96 | 1 | -0.26 |
| IGG1/FcγR III | KC128A | 1.02 | 1 | -0.06 |
| IGG1/FcγR III | DC129A | -0.46 | 1 | -0.07 |
| IGG1/FcγR III | RC130A | 1.85 | 1 | -1.72 |
| IGG1/FcγR III | RC130T | 1.48 | 1 | -0.66 |
| IGG1/FcγR III | KC131T | 0.76 | 1 | -0.31 |
| IGG1/FcγR III | YC132A | 1.56 | 1 | -0.73 |
| IGG1/FcγR III | AC144S | -0.27 | 1 | 0.00 |
| IGG1/FcγR III | TC145A | -0.34 | 1 | 0.00 |
| IGG1/FcγR III | SC149A | -0.39 | 1 | 0.00 |
| IGG1/FcγR III | CC150A | 1.36 | 1 | 0.00 |
| IGG1/FcγR III | SC151A | -0.08 | 1 | 0.00 |
| IGG1/FcγR III | SC160A | -0.20 | 1 | 0.14 |
| IGG1/FcγR N | A238A | -0.24 | 1 | -0.03 |
| IGG1/FcγR N | DA265A | -0.12 | 1 | 0.00 |
| IGG1/FcγR N | NA297A | 0.13 | 1 | 0.00 |
| IGG1/FcγR N | AA327Q | 0.02 | 1 | 0.00 |
| IGG1/FcγR N | PA329A | 0.13 | 1 | 0.00 |
| IGG1/FcγR N | DA270A | -0.03 | 1 | 0.00 |
| IGG1/FcγR N | QA295A | 0.14 | 1 | 0.00 |
| IGG1/FcγR N | TA256A | -0.38 | 1 | -0.87 |
| IGG1/FcγR N | KA290A | 0.14 | 1 | 0.00 |
| IGG1/FcγR N | RA255A | 0.31 | 1 | -1.71 |
| IGG1/FcγR N | EA258A | -0.10 | 1 | -0.09 |
| IGG1/FcγR N | SA267A | -0.05 | 1 | 0.00 |
| IGG1/FcγR N | EA272A | -0.17 | 1 | 0.00 |
| IGG1/FcγR N | NA276A | -0.08 | 1 | 0.00 |
| IGG1/FcγR N | DA280A | 0.12 | 1 | -0.04 |
| IGG1/FcγR N | HA285A | 0.10 | 1 | 0.00 |
| IGG1/FcγR N | NA286A | -0.13 | 1 | 0.00 |
| IGG1/FcγR N | TA307A | -0.35 | 1 | 0.15 |
| IGG1/FcγR N | LA309A | 0.27 | 1 | 0.43 |
| IGG1/FcγR N | NA315A | 0.16 | 1 | -0.01 |
| IGG1/FcγR N | KA326A | -0.02 | 1 | 0.00 |
| IGG1/FcγR N | PA331A | 0.10 | 1 | 0.00 |
| IGG1/FcγR N | SA337A | -0.02 | 1 | 0.00 |
| IGG1/FcγR N | AA378Q | -0.16 | 1 | 0.06 |
| IGG1/FcγR N | EA430A | 0.04 | 1 | -0.10 |
| IGG1/FcγR N | HA268A | -0.01 | 1 | 0.00 |
| IGG1/FcγR N | RA301A | 0.09 | 1 | 0.00 |
| IGG1/FcγR N | KA322A | 0.01 | 1 | 0.00 |
| IGG1/FcγR N | RA292A | 0.12 | 1 | 0.00 |
| IGG1/FcγR N | KA414A | -0.01 | 1 | 0.00 |
| IGG1/FcγR N | SA298A | 0.13 | 1 | 0.00 |
| IGG1/FcγR N | SA239A | -0.03 | 1 | 0.00 |
| IGG1/FcγR N | EA269A | -0.03 | 1 | 0.00 |
| IGG1/FcγR N | EA293A | 0.10 | 1 | 0.00 |
| IGG1/FcγR N | YA296F | 0.14 | 1 | -0.04 |
| IGG1/FcγR N | VA303A | -0.14 | 1 | 0.00 |
| IGG1/FcγR N | KA338A | -0.08 | 1 | 0.14 |
| IGG1/FcγR N | DA376A | -0.22 | 1 | 0.02 |
| IGG1/FcγR N | EA333A | -0.02 | 1 | 0.00 |
| IGG1/FcγR N | KA334A | -0.03 | 1 | 0.00 |
| IGG1/FcγR N | KA288A | 0.57 | 1 | 0.06 |
| IGG1/FcγR N | VA305A | -0.22 | 1 | 0.00 |
| IGG1/FcγR N | QA311A | -0.29 | 1 | 0.01 |
| IGG1/FcγR N | DA312A | -0.24 | 1 | -0.29 |
| IGG1/FcγR N | KA317A | -0.22 | 1 | -0.03 |
| IGG1/FcγR N | KA360A | -0.16 | 1 | 0.00 |
| IGG1/FcγR N | QA362A | -0.13 | 1 | 0.00 |
| IGG1/FcγR N | EA380A | -0.46 | 1 | -0.02 |
| IGG1/FcγR N | EA382A | -0.24 | 1 | 0.01 |
| IGG1/FcγR N | SA415A | 0.49 | 1 | 0.00 |
| IGG1/FcγR N | SA424A | -0.20 | 1 | 0.00 |
| IGG1/FcγR N | HA433A | 0.53 | 1 | -1.28 |
| IGG1/FcγR N | NA434A | -0.74 | 1 | -0.03 |

Table S2

Sub-group 2 of the validation dataset. Single- and double-free models. Bound (template) structures were homologous of the free structures (rather than being the same protein). ZEMu vs. experimental ΔΔG. Mutant nomenclature: same as table S1.

| **MODEL** | **Mutant** | **ΔΔGexp (kcal/mol)** | **number of substitutions** | **ΔΔG ZEMu (kcal/mol)** |
| --- | --- | --- | --- | --- |
| Single-free template model of IgG1/FcγRI (based on IgG1/FcγRIII crystal complex) | EA233P,EB233P | 1,26 | 2 | 0,71 |
| Single-free template model of IgG1/FcγRI (based on IgG1/FcγRIII crystal complex) | PA238A,PB238A | 0,3 | 2 | 1,77 |
| Single-free template model of IgG1/FcγRI (based on IgG1/FcγRIII crystal complex) | DA265A,DB265A | 1,09 | 2 | 0,74 |
| Single-free template model of IgG1/FcγRI (based on IgG1/FcγRIII crystal complex) | NA297A,NB297A | 1,12 | 2 | -0,01 |
| Single-free template model of IgG1/FcγRI (based on IgG1/FcγRIII crystal complex) | AA327Q,AB327Q | 0,3 | 2 | 1,85 |
| Single-free template model of IgG1/FcγRI (based on IgG1/FcγRIII crystal complex) | PA329A,PB329A | 0,43 | 2 | 1,58 |
| Single-free template model of IgG1/FcγRI (based on IgG1/FcγRIII crystal complex) | DA270A,DB270A | 0,16 | 2 | 0,27 |
| Single-free template model of IgG1/FcγRI (based on IgG1/FcγRIII crystal complex) | QA295A,QB295A | 0 | 2 | -0,47 |
| Single-free template model of IgG1/FcγRI (based on IgG1/FcγRIII crystal complex) | AA327S,AB327S | 0,09 | 2 | -0,57 |
| Single-free template model of IgG1/FcγRI (based on IgG1/FcγRIII crystal complex) | TA256A,TB256A | -0,01 | 2 | 0 |
| Single-free template model of IgG1/FcγRI (based on IgG1/FcγRIII crystal complex) | KA290A,KB290A | -0,01 | 2 | -0,01 |
| Single-free template model of IgG1/FcγRI (based on IgG1/FcγRIII crystal complex) | RA255A,RB255A | 0,01 | 2 | 0 |
| Single-free template model of IgG1/FcγRI (based on IgG1/FcγRIII crystal complex) | EA258A,EB258A | -0,1 | 2 | 0 |
| Single-free template model of IgG1/FcγRI (based on IgG1/FcγRIII crystal complex) | SA267A,SB267A | -0,05 | 2 | 1,1 |
| Single-free template model of IgG1/FcγRI (based on IgG1/FcγRIII crystal complex) | EA272A,EB272A | -0,03 | 2 | -0,08 |
| Single-free template model of IgG1/FcγRI (based on IgG1/FcγRIII crystal complex) | NA276A,NB276A | -0,03 | 2 | 0 |
| Single-free template model of IgG1/FcγRI (based on IgG1/FcγRIII crystal complex) | DA280A,DB280A | -0,02 | 2 | 0 |
| Single-free template model of IgG1/FcγRI (based on IgG1/FcγRIII crystal complex) | HA285A,HB285A | 0,02 | 2 | 0 |
| Single-free template model of IgG1/FcγRI (based on IgG1/FcγRIII crystal complex) | NA286A,NB286A | 0,03 | 2 | 0 |
| Single-free template model of IgG1/FcγRI (based on IgG1/FcγRIII crystal complex) | TA307A,TB307A | 0,01 | 2 | 0 |
| Single-free template model of IgG1/FcγRI (based on IgG1/FcγRIII crystal complex) | LA309A,LB309A | 0,04 | 2 | 0 |
| Single-free template model of IgG1/FcγRI (based on IgG1/FcγRIII crystal complex) | NA315A,NB315A | 0,01 | 2 | 0 |
| Single-free template model of IgG1/FcγRI (based on IgG1/FcγRIII crystal complex) | KA326A,KB326A | -0,02 | 2 | 0,37 |
| Single-free template model of IgG1/FcγRI (based on IgG1/FcγRIII crystal complex) | PA331A,PB331A | -0,01 | 2 | 0,89 |
| Single-free template model of IgG1/FcγRI (based on IgG1/FcγRIII crystal complex) | SA337A,SB337A | -0,09 | 2 | 0 |
| Single-free template model of IgG1/FcγRI (based on IgG1/FcγRIII crystal complex) | AA378Q,AB378Q | -0,03 | 2 | 0 |
| Single-free template model of IgG1/FcγRI (based on IgG1/FcγRIII crystal complex) | EA430A,EB430A | -0,03 | 2 | 0 |
| Single-free template model of IgG1/FcγRI (based on IgG1/FcγRIII crystal complex) | HA268A,HB268A | -0,05 | 2 | 0,03 |
| Single-free template model of IgG1/FcγRI (based on IgG1/FcγRIII crystal complex) | RA301A,RB301A | -0,03 | 2 | -0,67 |
| Single-free template model of IgG1/FcγRI (based on IgG1/FcγRIII crystal complex) | KA322A,KB322A | 0,04 | 2 | -0,04 |
| Single-free template model of IgG1/FcγRI (based on IgG1/FcγRIII crystal complex) | RA292A,RB292A | 0,03 | 2 | -0,02 |
| Single-free template model of IgG1/FcγRI (based on IgG1/FcγRIII crystal complex) | KA414A,KB414A | 0 | 2 | 0 |
| Single-free template model of IgG1/FcγRI (based on IgG1/FcγRIII crystal complex) | SA298A,SB298A | -0,06 | 2 | -0,42 |
| Single-free template model of IgG1/FcγRI (based on IgG1/FcγRIII crystal complex) | SA239A,SB239A | 0,12 | 2 | 0,68 |
| Single-free template model of IgG1/FcγRI (based on IgG1/FcγRIII crystal complex) | EA269A,EB269A | 0,29 | 2 | 2,08 |
| Single-free template model of IgG1/FcγRI (based on IgG1/FcγRIII crystal complex) | EA293A,EB293A | -0,06 | 2 | 0 |
| Single-free template model of IgG1/FcγRI (based on IgG1/FcγRIII crystal complex) | YA296F,YB296F | -0,02 | 2 | -0,05 |
| Single-free template model of IgG1/FcγRI (based on IgG1/FcγRIII crystal complex) | VA303A,VB303A | 0,06 | 2 | -0,03 |
| Single-free template model of IgG1/FcγRI (based on IgG1/FcγRIII crystal complex) | AA327G,AB327G | 0,02 | 2 | -0,16 |
| Single-free template model of IgG1/FcγRI (based on IgG1/FcγRIII crystal complex) | KA338A,KB338A | 0,06 | 2 | 0 |
| Single-free template model of IgG1/FcγRI (based on IgG1/FcγRIII crystal complex) | DA376A,DB376A | 0 | 2 | 0 |
| Single-free template model of IgG1/FcγRI (based on IgG1/FcγRIII crystal complex) | EA333A,EB333A | 0,01 | 2 | -0,07 |
| Single-free template model of IgG1/FcγRI (based on IgG1/FcγRIII crystal complex) | KA334A,KB334A | -0,03 | 2 | -0,09 |
| Single-free template model of IgG1/FcγRI (based on IgG1/FcγRIII crystal complex) | AA339T,AB339T | -0,03 | 2 | 0 |
| Single-free template model of IgG1/FcγRI (based on IgG1/FcγRIII crystal complex) | IA253A,IB253A | 0,02 | 2 | 0 |
| Single-free template model of IgG1/FcγRI (based on IgG1/FcγRIII crystal complex) | SA254A,SB254A | 0,02 | 2 | 0 |
| Single-free template model of IgG1/FcγRI (based on IgG1/FcγRIII crystal complex) | KA288A,KB288A | 0,08 | 2 | 0 |
| Single-free template model of IgG1/FcγRI (based on IgG1/FcγRIII crystal complex) | VA305A,VB305A | -0,02 | 2 | 0 |
| Single-free template model of IgG1/FcγRI (based on IgG1/FcγRIII crystal complex) | QA311A,QB311A | 0,04 | 2 | 0 |
| Single-free template model of IgG1/FcγRI (based on IgG1/FcγRIII crystal complex) | DA312A,DB312A | -0,01 | 2 | 0 |
| Single-free template model of IgG1/FcγRI (based on IgG1/FcγRIII crystal complex) | KA317A,KB317A | 0,05 | 2 | 0 |
| Single-free template model of IgG1/FcγRI (based on IgG1/FcγRIII crystal complex) | KA360A,KB360A | -0,01 | 2 | 0,11 |
| Single-free template model of IgG1/FcγRI (based on IgG1/FcγRIII crystal complex) | QA362A,QB362A | 0 | 2 | 0 |
| Single-free template model of IgG1/FcγRI (based on IgG1/FcγRIII crystal complex) | EA380A,EB380A | -0,02 | 2 | 0 |
| Single-free template model of IgG1/FcγRI (based on IgG1/FcγRIII crystal complex) | EA382A,EB382A | -0,03 | 2 | 0 |
| Single-free template model of IgG1/FcγRI (based on IgG1/FcγRIII crystal complex) | SA415A,SB415A | -0,02 | 2 | 0 |
| Single-free template model of IgG1/FcγRI (based on IgG1/FcγRIII crystal complex) | SA424A,SB424A | 0,01 | 2 | 0 |
| Single-free template model of IgG1/FcγRI (based on IgG1/FcγRIII crystal complex) | HA433A,HB433A | 0,01 | 2 | 0 |
| Single-free template model of IgG1/FcγRI (based on IgG1/FcγRIII crystal complex) | NA434A,NB434A | 0 | 2 | 0 |
| Single-free template model of IgG1/FcγRI (based on IgG1/FcγRIII crystal complex) | HA435A,HB435A | -0,13 | 2 | 0 |
| Single-free template model of IgG1/FcγRI (based on IgG1/FcγRIII crystal complex) | YA436A,YB436A | 0,01 | 2 | 0 |
| Single-free template model of IgG1/FcγRI (based on IgG1/FcγRIII crystal complex) | GA236A,GB236A | 1,21 | 2 | 4,86 |
| Single-free template model of IgG1/FcγRI (based on IgG1/FcγRIII crystal complex) | IA332E,IB332E | -0,66 | 2 | -0,03 |
| Single-free template model of IgG1/FcγRI (based on IgG1/FcγRIII crystal complex) | IA332E,IA332E,GA236A,IB332E,IB332E,GB236A | 0 | 6 | 5,06 |
| Single-free template model of IgG1/FcγRI (based on IgG1/FcγRIII crystal complex) | SA239D,SA239D,IA332E,SB239D,SB239D,IB332E | -1,1 | 6 | -0,69 |
| Single-free template model of IgG1/FcγRI (based on IgG1/FcγRIII crystal complex) | SA239D,SA239D,IA332E,GA236A,SB239D,SB239D,IB332E,GB236A | -0,62 | 8 | 0,64 |
| Double-free template model IgG1/FcγRI (based on IgG1/FcγRIII crystal complex) | PA238A,PB238A | 0,3 | 2 | 0,9 |
| Double-free template model IgG1/FcγRI (based on IgG1/FcγRIII crystal complex) | DA265A,DB265A | 1,09 | 2 | 0,66 |
| Double-free template model IgG1/FcγRI (based on IgG1/FcγRIII crystal complex) | NA297A,NB297A | 1,12 | 2 | 0,99 |
| Double-free template model IgG1/FcγRI (based on IgG1/FcγRIII crystal complex) | AA327Q,AB327Q | 0,3 | 2 | 0,38 |
| Double-free template model IgG1/FcγRI (based on IgG1/FcγRIII crystal complex) | PA329A,PB329A | 0,43 | 2 | 3,17 |
| Double-free template model IgG1/FcγRI (based on IgG1/FcγRIII crystal complex) | DA270A,DB270A | 0,16 | 2 | 1,8 |
| Double-free template model IgG1/FcγRI (based on IgG1/FcγRIII crystal complex) | QA295A,QB295A | 0 | 2 | -0,34 |
| Double-free template model IgG1/FcγRI (based on IgG1/FcγRIII crystal complex) | AA327S,AB327S | 0,09 | 2 | -0,38 |
| Double-free template model IgG1/FcγRI (based on IgG1/FcγRIII crystal complex) | TA256A,TB256A | -0,01 | 2 | 0 |
| Double-free template model IgG1/FcγRI (based on IgG1/FcγRIII crystal complex) | KA290A,KB290A | -0,01 | 2 | 0,01 |
| Double-free template model IgG1/FcγRI (based on IgG1/FcγRIII crystal complex) | RA255A,RB255A | 0,01 | 2 | 0 |
| Double-free template model IgG1/FcγRI (based on IgG1/FcγRIII crystal complex) | EA258A,EB258A | -0,1 | 2 | 0 |
| Double-free template model IgG1/FcγRI (based on IgG1/FcγRIII crystal complex) | SA267A,SB267A | -0,05 | 2 | -0,49 |
| Double-free template model IgG1/FcγRI (based on IgG1/FcγRIII crystal complex) | EA272A,EB272A | -0,03 | 2 | 0,04 |
| Double-free template model IgG1/FcγRI (based on IgG1/FcγRIII crystal complex) | NA276A,NB276A | -0,03 | 2 | -0,03 |
| Double-free template model IgG1/FcγRI (based on IgG1/FcγRIII crystal complex) | DA280A,DB280A | -0,02 | 2 | 0 |
| Double-free template model IgG1/FcγRI (based on IgG1/FcγRIII crystal complex) | HA285A,HB285A | 0,02 | 2 | -0,01 |
| Double-free template model IgG1/FcγRI (based on IgG1/FcγRIII crystal complex) | NA286A,NB286A | 0,03 | 2 | 0,36 |
| Double-free template model IgG1/FcγRI (based on IgG1/FcγRIII crystal complex) | TA307A,TB307A | 0,01 | 2 | -0,37 |
| Double-free template model IgG1/FcγRI (based on IgG1/FcγRIII crystal complex) | LA309A,LB309A | 0,04 | 2 | 0 |
| Double-free template model IgG1/FcγRI (based on IgG1/FcγRIII crystal complex) | NA315A,NB315A | 0,01 | 2 | 0 |
| Double-free template model IgG1/FcγRI (based on IgG1/FcγRIII crystal complex) | KA326A,KB326A | -0,02 | 2 | 0,44 |
| Double-free template model IgG1/FcγRI (based on IgG1/FcγRIII crystal complex) | PA331A,PB331A | -0,01 | 2 | -0,32 |
| Double-free template model IgG1/FcγRI (based on IgG1/FcγRIII crystal complex) | SA337A,SB337A | -0,09 | 2 | 0 |
| Double-free template model IgG1/FcγRI (based on IgG1/FcγRIII crystal complex) | AA378Q,AB378Q | -0,03 | 2 | 0 |
| Double-free template model IgG1/FcγRI (based on IgG1/FcγRIII crystal complex) | EA430A,EB430A | -0,03 | 2 | 0 |
| Double-free template model IgG1/FcγRI (based on IgG1/FcγRIII crystal complex) | HA268A,HB268A | -0,05 | 2 | -0,1 |
| Double-free template model IgG1/FcγRI (based on IgG1/FcγRIII crystal complex) | RA301A,RB301A | -0,03 | 2 | 0,01 |
| Double-free template model IgG1/FcγRI (based on IgG1/FcγRIII crystal complex) | KA322A,KB322A | 0,04 | 2 | -0,02 |
| Double-free template model IgG1/FcγRI (based on IgG1/FcγRIII crystal complex) | RA292A,RB292A | 0,03 | 2 | -0,07 |
| Double-free template model IgG1/FcγRI (based on IgG1/FcγRIII crystal complex) | KA414A,KB414A | 0 | 2 | 0,36 |
| Double-free template model IgG1/FcγRI (based on IgG1/FcγRIII crystal complex) | SA298A,SB298A | -0,06 | 2 | -0,5 |
| Double-free template model IgG1/FcγRI (based on IgG1/FcγRIII crystal complex) | SA239A,SB239A | 0,12 | 2 | -1,61 |
| Double-free template model IgG1/FcγRI (based on IgG1/FcγRIII crystal complex) | EA269A,EB269A | 0,29 | 2 | 1,94 |
| Double-free template model IgG1/FcγRI (based on IgG1/FcγRIII crystal complex) | EA293A,EB293A | -0,06 | 2 | 0,19 |
| Double-free template model IgG1/FcγRI (based on IgG1/FcγRIII crystal complex) | YA296F,YB296F | -0,02 | 2 | -0,4 |
| Double-free template model IgG1/FcγRI (based on IgG1/FcγRIII crystal complex) | VA303A,VB303A | 0,06 | 2 | 0 |
| Double-free template model IgG1/FcγRI (based on IgG1/FcγRIII crystal complex) | AA327G,AB327G | 0,02 | 2 | -0,17 |
| Double-free template model IgG1/FcγRI (based on IgG1/FcγRIII crystal complex) | KA338A,KB338A | 0,06 | 2 | 0,37 |
| Double-free template model IgG1/FcγRI (based on IgG1/FcγRIII crystal complex) | DA376A,DB376A | 0 | 2 | 0 |
| Double-free template model IgG1/FcγRI (based on IgG1/FcγRIII crystal complex) | EA333A,EB333A | 0,01 | 2 | 0,07 |
| Double-free template model IgG1/FcγRI (based on IgG1/FcγRIII crystal complex) | KA334A,KB334A | -0,03 | 2 | -0,01 |
| Double-free template model IgG1/FcγRI (based on IgG1/FcγRIII crystal complex) | AA339T,AB339T | -0,03 | 2 | 0 |
| Double-free template model IgG1/FcγRI (based on IgG1/FcγRIII crystal complex) | IA253A,IB253A | 0,02 | 2 | -0,37 |
| Double-free template model IgG1/FcγRI (based on IgG1/FcγRIII crystal complex) | SA254A,SB254A | 0,02 | 2 | 0 |
| Double-free template model IgG1/FcγRI (based on IgG1/FcγRIII crystal complex) | KA288A,KB288A | 0,08 | 2 | 0 |
| Double-free template model IgG1/FcγRI (based on IgG1/FcγRIII crystal complex) | VA305A,VB305A | -0,02 | 2 | 0,37 |
| Double-free template model IgG1/FcγRI (based on IgG1/FcγRIII crystal complex) | QA311A,QB311A | 0,04 | 2 | 0 |
| Double-free template model IgG1/FcγRI (based on IgG1/FcγRIII crystal complex) | DA312A,DB312A | -0,01 | 2 | 0,37 |
| Double-free template model IgG1/FcγRI (based on IgG1/FcγRIII crystal complex) | KA317A,KB317A | 0,05 | 2 | 0 |
| Double-free template model IgG1/FcγRI (based on IgG1/FcγRIII crystal complex) | KA360A,KB360A | -0,01 | 2 | -0,39 |
| Double-free template model IgG1/FcγRI (based on IgG1/FcγRIII crystal complex) | QA362A,QB362A | 0 | 2 | -0,25 |
| Double-free template model IgG1/FcγRI (based on IgG1/FcγRIII crystal complex) | EA380A,EB380A | -0,02 | 2 | 0 |
| Double-free template model IgG1/FcγRI (based on IgG1/FcγRIII crystal complex) | EA382A,EB382A | -0,03 | 2 | 0 |
| Double-free template model IgG1/FcγRI (based on IgG1/FcγRIII crystal complex) | SA415A,SB415A | -0,02 | 2 | 0 |
| Double-free template model IgG1/FcγRI (based on IgG1/FcγRIII crystal complex) | SA424A,SB424A | 0,01 | 2 | 0 |
| Double-free template model IgG1/FcγRI (based on IgG1/FcγRIII crystal complex) | HA433A,HB433A | 0,01 | 2 | 0 |
| Double-free template model IgG1/FcγRI (based on IgG1/FcγRIII crystal complex) | NA434A,NB434A | 0 | 2 | 0 |
| Double-free template model IgG1/FcγRI (based on IgG1/FcγRIII crystal complex) | HA435A,HB435A | -0,13 | 2 | 0 |
| Double-free template model IgG1/FcγRI (based on IgG1/FcγRIII crystal complex) | YA436A,YB436A | 0,01 | 2 | 0 |
| Double-free template model IgG1/FcγRI (based on IgG1/FcγRIII crystal complex) | IA332E,IB332E | -0,66 | 2 | 0,32 |
| Double-free template model IgG1/FcγRI (based on IgG1/FcγRIII crystal complex) | SA239D,SA239D,IA332E,SB239D,SB239D,IB332E | -1,1 | 6 | -1,36 |
| Fitted model IgG1/FcγR I | PA238A,PB238A | 0,3 | 2 | 0,23 |
| Fitted model IgG1/FcγR I | DA265A,DB265A | 1,09 | 2 | 0,55 |
| Fitted model IgG1/FcγR I | NA297A,NB297A | 1,12 | 2 | 0,18 |
| Fitted model IgG1/FcγR I | AA327Q,AB327Q | 0,3 | 2 |  |
| Fitted model IgG1/FcγR I | PA329A,PB329A | 0,43 | 2 | 1,94 |
| Fitted model IgG1/FcγR I | DA270A,DB270A | 0,16 | 2 | 1,75 |
| Fitted model IgG1/FcγR I | QA295A,QB295A | 0 | 2 | -0,71 |
| Fitted model IgG1/FcγR I | AA327S,AB327S | 0,09 | 2 | 0,2 |
| Fitted model IgG1/FcγR I | TA256A,TB256A | -0,01 | 2 | 0 |
| Fitted model IgG1/FcγR I | KA290A,KB290A | -0,01 | 2 | 0 |
| Fitted model IgG1/FcγR I | RA255A,RB255A | 0,01 | 2 | 0 |
| Fitted model IgG1/FcγR I | EA258A,EB258A | -0,1 | 2 | 0 |
| Fitted model IgG1/FcγR I | SA267A,SB267A | -0,05 | 2 | -0,35 |
| Fitted model IgG1/FcγR I | EA272A,EB272A | -0,03 | 2 | -0,05 |
| Fitted model IgG1/FcγR I | NA276A,NB276A | -0,03 | 2 | 0,01 |
| Fitted model IgG1/FcγR I | DA280A,DB280A | -0,02 | 2 | 0 |
| Fitted model IgG1/FcγR I | HA285A,HB285A | 0,02 | 2 | 0 |
| Fitted model IgG1/FcγR I | NA286A,NB286A | 0,03 | 2 | 0 |
| Fitted model IgG1/FcγR I | TA307A,TB307A | 0,01 | 2 | 0 |
| Fitted model IgG1/FcγR I | LA309A,LB309A | 0,04 | 2 | 0 |
| Fitted model IgG1/FcγR I | NA315A,NB315A | 0,01 | 2 | 0 |
| Fitted model IgG1/FcγR I | KA326A,KB326A | -0,02 | 2 | -0,19 |
| Fitted model IgG1/FcγR I | PA331A,PB331A | -0,01 | 2 | -0,83 |
| Fitted model IgG1/FcγR I | SA337A,SB337A | -0,09 | 2 | 0 |
| Fitted model IgG1/FcγR I | AA378Q,AB378Q | -0,03 | 2 | -0,05 |
| Fitted model IgG1/FcγR I | EA430A,EB430A | -0,03 | 2 | 0,01 |
| Fitted model IgG1/FcγR I | HA268A,HB268A | -0,05 | 2 | -0,93 |
| Fitted model IgG1/FcγR I | RA301A,RB301A | -0,03 | 2 | -0,41 |
| Fitted model IgG1/FcγR I | KA322A,KB322A | 0,04 | 2 | -0,04 |
| Fitted model IgG1/FcγR I | RA292A,RB292A | 0,03 | 2 | -0,85 |
| Fitted model IgG1/FcγR I | KA414A,KB414A | 0 | 2 | 0 |
| Fitted model IgG1/FcγR I | SA298A,SB298A | -0,06 | 2 | -0,42 |
| Fitted model IgG1/FcγR I | SA239A,SB239A | 0,12 | 2 | -0,38 |
| Fitted model IgG1/FcγR I | EA269A,EB269A | 0,29 | 2 | -0,21 |
| Fitted model IgG1/FcγR I | EA293A,EB293A | -0,06 | 2 | -1,34 |
| Fitted model IgG1/FcγR I | YA296F,YB296F | -0,02 | 2 | 0,47 |
| Fitted model IgG1/FcγR I | VA303A,VB303A | 0,06 | 2 | 0 |
| Fitted model IgG1/FcγR I | AA327G,AB327G | 0,02 | 2 | -0,29 |
| Fitted model IgG1/FcγR I | KA338A,KB338A | 0,06 | 2 | 0 |
| Fitted model IgG1/FcγR I | DA376A,DB376A | 0 | 2 | 0 |
| Fitted model IgG1/FcγR I | EA333A,EB333A | 0,01 | 2 | 0,02 |
| Fitted model IgG1/FcγR I | KA334A,KB334A | -0,03 | 2 | 0,05 |
| Fitted model IgG1/FcγR I | AA339T,AB339T | -0,03 | 2 | 0 |
| Fitted model IgG1/FcγR I | IA253A,IB253A | 0,02 | 2 | 0 |
| Fitted model IgG1/FcγR I | SA254A,SB254A | 0,02 | 2 | 0 |
| Fitted model IgG1/FcγR I | KA288A,KB288A | 0,08 | 2 | -0,19 |
| Fitted model IgG1/FcγR I | VA305A,VB305A | -0,02 | 2 | 0 |
| Fitted model IgG1/FcγR I | QA311A,QB311A | 0,04 | 2 | 0 |
| Fitted model IgG1/FcγR I | DA312A,DB312A | -0,01 | 2 | 0 |
| Fitted model IgG1/FcγR I | KA317A,KB317A | 0,05 | 2 | 0 |
| Fitted model IgG1/FcγR I | KA360A,KB360A | -0,01 | 2 | -0,17 |
| Fitted model IgG1/FcγR I | QA362A,QB362A | 0 | 2 | 0 |
| Fitted model IgG1/FcγR I | EA380A,EB380A | -0,02 | 2 | 0,01 |
| Fitted model IgG1/FcγR I | EA382A,EB382A | -0,03 | 2 | 0 |
| Fitted model IgG1/FcγR I | SA415A,SB415A | -0,02 | 2 | 0 |
| Fitted model IgG1/FcγR I | SA424A,SB424A | 0,01 | 2 | 0 |
| Fitted model IgG1/FcγR I | HA433A,HB433A | 0,01 | 2 | 0,01 |
| Fitted model IgG1/FcγR I | NA434A,NB434A | 0 | 2 | 0 |
| Fitted model IgG1/FcγR I | HA435A,HB435A | -0,13 | 2 | 0 |
| Fitted model IgG1/FcγR I | YA436A,YB436A | 0,01 | 2 | 0 |
| Fitted model IgG1/FcγR I | IA332E,IB332E | -0,66 | 2 | 0,05 |
| Fitted model IgG1/FcγR I | SA239D,SA239D,IA332E,SB239D,SB239D,IB332E | -1,1 | 6 | -2,12 |
| Double-free template model IgG1/FcγRII (based on IgG1/FcγRIII crystal complex) | PA238A,PB238A | 0,57 | 2 | 0,14 |
| Double-free template model IgG1/FcγRII (based on IgG1/FcγRIII crystal complex) | DA265A,DB265A | 1,57 | 2 | 1,23 |
| Double-free template model IgG1/FcγRII (based on IgG1/FcγRIII crystal complex) | NA297A,NB297A | 1,77 | 2 | -4,65 |
| Double-free template model IgG1/FcγRII (based on IgG1/FcγRIII crystal complex) | AA327Q,AB327Q | 1,21 | 2 | -0,32 |
| Double-free template model IgG1/FcγRII (based on IgG1/FcγRIII crystal complex) | PA329A,PB329A | 1,5 | 2 | 0,98 |
| Double-free template model IgG1/FcγRII (based on IgG1/FcγRIII crystal complex) | DA270A,DB270A | 1,67 | 2 | -0,81 |
| Double-free template model IgG1/FcγRII (based on IgG1/FcγRIII crystal complex) | QA295A,QB295A | 0,28 | 2 | 1,06 |
| Double-free template model IgG1/FcγRII (based on IgG1/FcγRIII crystal complex) | AA327S,AB327S | 0,87 | 2 | 0,74 |
| Double-free template model IgG1/FcγRII (based on IgG1/FcγRIII crystal complex) | TA256A,TB256A | -0,2 | 2 | 0,00 |
| Double-free template model IgG1/FcγRII (based on IgG1/FcγRIII crystal complex) | KA290A,KB290A | -0,16 | 2 | 0,01 |
| Double-free template model IgG1/FcγRII (based on IgG1/FcγRIII crystal complex) | RA255A,RB255A | -0,16 | 2 | 0,00 |
| Double-free template model IgG1/FcγRII (based on IgG1/FcγRIII crystal complex) | EA258A,EB258A | -0,17 | 2 | 0,00 |
| Double-free template model IgG1/FcγRII (based on IgG1/FcγRIII crystal complex) | SA267A,SB267A | -0,25 | 2 | 0,00 |
| Double-free template model IgG1/FcγRII (based on IgG1/FcγRIII crystal complex) | EA272A,EB272A | -0,12 | 2 | -0,77 |
| Double-free template model IgG1/FcγRII (based on IgG1/FcγRIII crystal complex) | NA276A,NB276A | -0,15 | 2 | 0,00 |
| Double-free template model IgG1/FcγRII (based on IgG1/FcγRIII crystal complex) | DA280A,DB280A | -0,17 | 2 | 0,00 |
| Double-free template model IgG1/FcγRII (based on IgG1/FcγRIII crystal complex) | HA285A,HB285A | -0,14 | 2 | 0,00 |
| Double-free template model IgG1/FcγRII (based on IgG1/FcγRIII crystal complex) | NA286A,NB286A | -0,13 | 2 | -0,02 |
| Double-free template model IgG1/FcγRII (based on IgG1/FcγRIII crystal complex) | TA307A,TB307A | -0,04 | 2 | 0,00 |
| Double-free template model IgG1/FcγRII (based on IgG1/FcγRIII crystal complex) | LA309A,LB309A | -0,07 | 2 | 0,00 |
| Double-free template model IgG1/FcγRII (based on IgG1/FcγRIII crystal complex) | NA315A,NB315A | -0,08 | 2 | 0,00 |
| Double-free template model IgG1/FcγRII (based on IgG1/FcγRIII crystal complex) | KA326A,KB326A | -0,12 | 2 | -0,20 |
| Double-free template model IgG1/FcγRII (based on IgG1/FcγRIII crystal complex) | PA331A,PB331A | -0,15 | 2 | -1,21 |
| Double-free template model IgG1/FcγRII (based on IgG1/FcγRIII crystal complex) | SA337A,SB337A | -0,12 | 2 | 0,00 |
| Double-free template model IgG1/FcγRII (based on IgG1/FcγRIII crystal complex) | AA378Q,AB378Q | -0,2 | 2 | 0,00 |
| Double-free template model IgG1/FcγRII (based on IgG1/FcγRIII crystal complex) | EA430A,EB430A | -0,13 | 2 | 0,00 |
| Double-free template model IgG1/FcγRII (based on IgG1/FcγRIII crystal complex) | HA268A,HB268A | -0,11 | 2 | -1,43 |
| Double-free template model IgG1/FcγRII (based on IgG1/FcγRIII crystal complex) | RA301A,RB301A | -0,08 | 2 | 0,09 |
| Double-free template model IgG1/FcγRII (based on IgG1/FcγRIII crystal complex) | KA322A,KB322A | -0,09 | 2 | 0,00 |
| Double-free template model IgG1/FcγRII (based on IgG1/FcγRIII crystal complex) | RA292A,RB292A | 0,78 | 2 | 0,08 |
| Double-free template model IgG1/FcγRII (based on IgG1/FcγRIII crystal complex) | KA414A,KB414A | 0,26 | 2 | 0,00 |
| Double-free template model IgG1/FcγRII (based on IgG1/FcγRIII crystal complex) | SA298A,SB298A | 0,54 | 2 | 1,46 |
| Double-free template model IgG1/FcγRII (based on IgG1/FcγRIII crystal complex) | SA239A,SB239A | 0,19 | 2 | -0,03 |
| Double-free template model IgG1/FcγRII (based on IgG1/FcγRIII crystal complex) | EA269A,EB269A | 0,26 | 2 | -1,25 |
| Double-free template model IgG1/FcγRII (based on IgG1/FcγRIII crystal complex) | EA293A,EB293A | -0,05 | 2 | 0,31 |
| Double-free template model IgG1/FcγRII (based on IgG1/FcγRIII crystal complex) | YA296F,YB296F | 0,02 | 2 | -0,30 |
| Double-free template model IgG1/FcγRII (based on IgG1/FcγRIII crystal complex) | VA303A,VB303A | 0,09 | 2 | 0,00 |
| Double-free template model IgG1/FcγRII (based on IgG1/FcγRIII crystal complex) | AA327G,AB327G | 0,05 | 2 | -0,65 |
| Double-free template model IgG1/FcγRII (based on IgG1/FcγRIII crystal complex) | KA338A,KB338A | 0,15 | 2 | 0,00 |
| Double-free template model IgG1/FcγRII (based on IgG1/FcγRIII crystal complex) | DA376A,DB376A | 0,13 | 2 | 0,00 |
| Double-free template model IgG1/FcγRII (based on IgG1/FcγRIII crystal complex) | EA333A,EB333A | 0,05 | 2 | -0,01 |
| Double-free template model IgG1/FcγRII (based on IgG1/FcγRIII crystal complex) | KA334A,KB334A | -0,01 | 2 | 0,00 |
| Double-free template model IgG1/FcγRII (based on IgG1/FcγRIII crystal complex) | AA339T,AB339T | -0,05 | 2 | 0,00 |
| Double-free template model IgG1/FcγRII (based on IgG1/FcγRIII crystal complex) | IA253A,IB253A | -0,08 | 2 | 0,00 |
| Double-free template model IgG1/FcγRII (based on IgG1/FcγRIII crystal complex) | SA254A,SB254A | 0,02 | 2 | -0,02 |
| Double-free template model IgG1/FcγRII (based on IgG1/FcγRIII crystal complex) | KA288A,KB288A | -0,08 | 2 | 0,00 |
| Double-free template model IgG1/FcγRII (based on IgG1/FcγRIII crystal complex) | VA305A,VB305A | -0,07 | 2 | 0,00 |
| Double-free template model IgG1/FcγRII (based on IgG1/FcγRIII crystal complex) | QA311A,QB311A | -0,06 | 2 | 0,00 |
| Double-free template model IgG1/FcγRII (based on IgG1/FcγRIII crystal complex) | DA312A,DB312A | -0,11 | 2 | 0,00 |
| Double-free template model IgG1/FcγRII (based on IgG1/FcγRIII crystal complex) | KA317A,KB317A | -0,07 | 2 | 0,00 |
| Double-free template model IgG1/FcγRII (based on IgG1/FcγRIII crystal complex) | KA360A,KB360A | -0,07 | 2 | 0,80 |
| Double-free template model IgG1/FcγRII (based on IgG1/FcγRIII crystal complex) | QA362A,QB362A | -0,02 | 2 | 0,00 |
| Double-free template model IgG1/FcγRII (based on IgG1/FcγRIII crystal complex) | EA380A,EB380A | -0,1 | 2 | 0,00 |
| Double-free template model IgG1/FcγRII (based on IgG1/FcγRIII crystal complex) | EA382A,EB382A | 0,03 | 2 | 0,00 |
| Double-free template model IgG1/FcγRII (based on IgG1/FcγRIII crystal complex) | SA415A,SB415A | 0,06 | 2 | 0,00 |
| Double-free template model IgG1/FcγRII (based on IgG1/FcγRIII crystal complex) | SA424A,SB424A | -0,02 | 2 | 0,00 |
| Double-free template model IgG1/FcγRII (based on IgG1/FcγRIII crystal complex) | HA433A,HB433A | 0,05 | 2 | 0,00 |
| Double-free template model IgG1/FcγRII (based on IgG1/FcγRIII crystal complex) | NA434A,NB434A | 0,02 | 2 | 0,00 |
| Double-free template model IgG1/FcγRII (based on IgG1/FcγRIII crystal complex) | HA435A,HB435A | 0,15 | 2 | 0,00 |
| Double-free template model IgG1/FcγRII (based on IgG1/FcγRIII crystal complex) | YA436A,YB436A | 0,04 | 2 | 0,00 |
| Double-free template model IgG1/FcγRII (based on IgG1/FcγRIII crystal complex) | GA236A,GB236A | -1,16 | 2 | 1,50 |
| Double-free template model IgG1/FcγRII (based on IgG1/FcγRIII crystal complex) | IA332E,IB332E | -0,15 | 2 | -0,92 |
| Double-free template model IgG1/FcγRII (based on IgG1/FcγRIII crystal complex) | IA332E,IB332E,GA236A,GB236A | -1,39 | 4 | 0,60 |
| Double-free template model IgG1/FcγRII (based on IgG1/FcγRIII crystal complex) | SA239D,SB239D,IA332E,IB332E | -0,81 | 4 | -0,93 |
| Double-free template model IgG1/FcγRII (based on IgG1/FcγRIII crystal complex) | SA239D,SB239D,IA332E,GA236A,IB332E,GB236A | -2,12 | 6 | 0,14 |
| Double-free template model IgG1/FcγRIII (based on IgG1/FcγRII crystal complex) | PA238A,PB238A | 1,57 | 2 | -0,85 |
| Double-free template model IgG1/FcγRIII (based on IgG1/FcγRII crystal complex) | DA265A,DB265A | 1,43 | 2 | 0,59 |
| Double-free template model IgG1/FcγRIII (based on IgG1/FcγRII crystal complex) | NA297A,NB297A | 2,08 | 2 | -1,88 |
| Double-free template model IgG1/FcγRIII (based on IgG1/FcγRII crystal complex) | AA327Q,AB327Q | 1,67 | 2 | 0,14 |
| Double-free template model IgG1/FcγRIII (based on IgG1/FcγRII crystal complex) | PA329A,PB329A | 0,92 | 2 | 0,02 |
| Double-free template model IgG1/FcγRIII (based on IgG1/FcγRII crystal complex) | DA270A,DB270A | 1,16 | 2 | 0,01 |
| Double-free template model IgG1/FcγRIII (based on IgG1/FcγRII crystal complex) | QA295A,QB295A | 0,82 | 2 | -0,67 |
| Double-free template model IgG1/FcγRIII (based on IgG1/FcγRII crystal complex) | AA327S,AB327S | 1,67 | 2 | 0,02 |
| Double-free template model IgG1/FcγRIII (based on IgG1/FcγRII crystal complex) | TA256A,TB256A | -0,16 | 2 | 0,00 |
| Double-free template model IgG1/FcγRIII (based on IgG1/FcγRII crystal complex) | KA290A,KB290A | -0,16 | 2 | 0,00 |
| Double-free template model IgG1/FcγRIII (based on IgG1/FcγRII crystal complex) | RA255A,RB255A | 0,01 | 2 | 0,00 |
| Double-free template model IgG1/FcγRIII (based on IgG1/FcγRII crystal complex) | EA258A,EB258A | -0,07 | 2 | 0,02 |
| Double-free template model IgG1/FcγRIII (based on IgG1/FcγRII crystal complex) | SA267A,SB267A | -0,03 | 2 | 0,44 |
| Double-free template model IgG1/FcγRIII (based on IgG1/FcγRII crystal complex) | EA272A,EB272A | 0,13 | 2 | -0,01 |
| Double-free template model IgG1/FcγRIII (based on IgG1/FcγRII crystal complex) | NA276A,NB276A | 0,03 | 2 | 0,02 |
| Double-free template model IgG1/FcγRIII (based on IgG1/FcγRII crystal complex) | DA280A,DB280A | -0,05 | 2 | 0,00 |
| Double-free template model IgG1/FcγRIII (based on IgG1/FcγRII crystal complex) | HA285A,HB285A | 0,08 | 2 | 0,00 |
| Double-free template model IgG1/FcγRIII (based on IgG1/FcγRII crystal complex) | NA286A,NB286A | -0,03 | 2 | 0,01 |
| Double-free template model IgG1/FcγRIII (based on IgG1/FcγRII crystal complex) | TA307A,TB307A | -0,05 | 2 | 0,00 |
| Double-free template model IgG1/FcγRIII (based on IgG1/FcγRII crystal complex) | LA309A,LB309A | -0,04 | 2 | -0,08 |
| Double-free template model IgG1/FcγRIII (based on IgG1/FcγRII crystal complex) | NA315A,NB315A | -0,04 | 2 | 0,00 |
| Double-free template model IgG1/FcγRIII (based on IgG1/FcγRII crystal complex) | KA326A,KB326A | -0,12 | 2 | 0,01 |
| Double-free template model IgG1/FcγRIII (based on IgG1/FcγRII crystal complex) | PA331A,PB331A | -0,05 | 2 | 0,11 |
| Double-free template model IgG1/FcγRIII (based on IgG1/FcγRII crystal complex) | SA337A,SB337A | 0,04 | 2 | 0,00 |
| Double-free template model IgG1/FcγRIII (based on IgG1/FcγRII crystal complex) | AA378Q,AB378Q | -0,1 | 2 | -0,02 |
| Double-free template model IgG1/FcγRIII (based on IgG1/FcγRII crystal complex) | EA430A,EB430A | -0,11 | 2 | 0,00 |
| Double-free template model IgG1/FcγRIII (based on IgG1/FcγRII crystal complex) | HA268A,HB268A | 0,36 | 2 | -6,56 |
| Double-free template model IgG1/FcγRIII (based on IgG1/FcγRII crystal complex) | RA301A,RB301A | 0,9 | 2 | -0,37 |
| Double-free template model IgG1/FcγRIII (based on IgG1/FcγRII crystal complex) | KA322A,KB322A | 0,28 | 2 | 0,10 |
| Double-free template model IgG1/FcγRIII (based on IgG1/FcγRII crystal complex) | RA292A,RB292A | 0,07 | 2 | 0,00 |
| Double-free template model IgG1/FcγRIII (based on IgG1/FcγRII crystal complex) | KA414A,KB414A | 0,12 | 2 | 0,00 |
| Double-free template model IgG1/FcγRIII (based on IgG1/FcγRII crystal complex) | SA298A,SB298A | -0,17 | 2 | -5,71 |
| Double-free template model IgG1/FcγRIII (based on IgG1/FcγRII crystal complex) | SA239A,SB239A | 0,8 | 2 | 0,42 |
| Double-free template model IgG1/FcγRIII (based on IgG1/FcγRII crystal complex) | EA269A,EB269A | 0,47 | 2 | 0,38 |
| Double-free template model IgG1/FcγRIII (based on IgG1/FcγRII crystal complex) | EA293A,EB293A | 0,69 | 2 | -0,06 |
| Double-free template model IgG1/FcγRIII (based on IgG1/FcγRII crystal complex) | YA296F,YB296F | 0,35 | 2 | -0,52 |
| Double-free template model IgG1/FcγRIII (based on IgG1/FcγRII crystal complex) | VA303A,VB303A | 0,66 | 2 | 0,08 |
| Double-free template model IgG1/FcγRIII (based on IgG1/FcγRII crystal complex) | AA327G,AB327G | 0,6 | 2 | -0,03 |
| Double-free template model IgG1/FcγRIII (based on IgG1/FcγRII crystal complex) | KA338A,KB338A | 1,12 | 2 | 0,00 |
| Double-free template model IgG1/FcγRIII (based on IgG1/FcγRII crystal complex) | DA376A,DB376A | 0,35 | 2 | 0,00 |
| Double-free template model IgG1/FcγRIII (based on IgG1/FcγRII crystal complex) | EA333A,EB333A | -0,14 | 2 | -0,15 |
| Double-free template model IgG1/FcγRIII (based on IgG1/FcγRII crystal complex) | KA334A,KB334A | -0,2 | 2 | -0,10 |
| Double-free template model IgG1/FcγRIII (based on IgG1/FcγRII crystal complex) | AA339T,AB339T | -0,17 | 2 | 0,00 |
| Double-free template model IgG1/FcγRIII (based on IgG1/FcγRII crystal complex) | IA253A,IB253A | -0,05 | 2 | 0,03 |
| Double-free template model IgG1/FcγRIII (based on IgG1/FcγRII crystal complex) | SA254A,SB254A | 0,19 | 2 | 0,00 |
| Double-free template model IgG1/FcγRIII (based on IgG1/FcγRII crystal complex) | KA288A,KB288A | -0,03 | 2 | 0,03 |
| Double-free template model IgG1/FcγRIII (based on IgG1/FcγRII crystal complex) | VA305A,VB305A | 0,1 | 2 | 0,00 |
| Double-free template model IgG1/FcγRIII (based on IgG1/FcγRII crystal complex) | QA311A,QB311A | 0,04 | 2 | -0,09 |
| Double-free template model IgG1/FcγRIII (based on IgG1/FcγRII crystal complex) | DA312A,DB312A | -0,12 | 2 | 0,00 |
| Double-free template model IgG1/FcγRIII (based on IgG1/FcγRII crystal complex) | KA317A,KB317A | -0,06 | 2 | 0,00 |
| Double-free template model IgG1/FcγRIII (based on IgG1/FcγRII crystal complex) | KA360A,KB360A | -0,12 | 2 | 0,00 |
| Double-free template model IgG1/FcγRIII (based on IgG1/FcγRII crystal complex) | QA362A,QB362A | -0,02 | 2 | 0,00 |
| Double-free template model IgG1/FcγRIII (based on IgG1/FcγRII crystal complex) | EA380A,EB380A | 0,05 | 2 | 0,00 |
| Double-free template model IgG1/FcγRIII (based on IgG1/FcγRII crystal complex) | EA382A,EB382A | 0,16 | 2 | 0,00 |
| Double-free template model IgG1/FcγRIII (based on IgG1/FcγRII crystal complex) | SA415A,SB415A | 0,09 | 2 | 0,00 |
| Double-free template model IgG1/FcγRIII (based on IgG1/FcγRII crystal complex) | SA424A,SB424A | 0,08 | 2 | 0,00 |
| Double-free template model IgG1/FcγRIII (based on IgG1/FcγRII crystal complex) | HA433A,HB433A | -0,01 | 2 | 0,00 |
| Double-free template model IgG1/FcγRIII (based on IgG1/FcγRII crystal complex) | NA434A,NB434A | 0,15 | 2 | 0,00 |
| Double-free template model IgG1/FcγRIII (based on IgG1/FcγRII crystal complex) | HA435A,HB435A | 0,15 | 2 | 0,00 |
| Double-free template model IgG1/FcγRIII (based on IgG1/FcγRII crystal complex) | YA436A,YB436A | 0,06 | 2 | 0,00 |
| Double-free template model IgG1/FcγRIII (based on IgG1/FcγRII crystal complex) | LC124Y | 0,68 | 1 | 0,20 |
| Double-free template model IgG1/FcγRIII (based on IgG1/FcγRII crystal complex) | QC125K | 0,43 | 1 | 0,02 |
| Double-free template model IgG1/FcγRIII (based on IgG1/FcγRII crystal complex) | NC126D | -0,03 | 1 | 0,00 |
| Double-free template model IgG1/FcγRIII (based on IgG1/FcγRII crystal complex) | FC153Y | 0,16 | 1 | 0,00 |
| Double-free template model IgG1/FcγRIII (based on IgG1/FcγRII crystal complex) | LC157K | -0,03 | 1 | -0,32 |
| Double-free template model IgG1/FcγRIII (based on IgG1/FcγRII crystal complex) | SC160Q | 0,11 | 1 | 0,57 |
| Double-free template model IgG1/FcγRIII (based on IgG1/FcγRII crystal complex) | KC161L | 0,55 | 1 | -0,57 |
| Double-free template model IgG1/FcγRIII (based on IgG1/FcγRII crystal complex) | NC162D | 0,12 | 1 | -0,31 |
| Double-free template model IgG1/FcγRIII (based on IgG1/FcγRII crystal complex) | SC164E | 0,15 | 1 | -0,11 |
| Double-free template model IgG1/FcγRIII (based on IgG1/FcγRII crystal complex) | EC36H | -0,1 | 1 | 0,00 |
| Double-free template model IgG1/FcγRIII (based on IgG1/FcγRII crystal complex) | DC37A | -0,31 | 1 | 0,00 |
| Double-free template model IgG1/FcγRIII (based on IgG1/FcγRII crystal complex) | SC66A | -0,2 | 1 | 0,00 |
| Double-free template model IgG1/FcγRIII (based on IgG1/FcγRII crystal complex) | SC79A | -0,11 | 1 | 0,06 |
| Double-free template model IgG1/FcγRIII (based on IgG1/FcγRII crystal complex) | DC80A | -0,29 | 1 | 0,00 |
| Double-free template model IgG1/FcγRIII (based on IgG1/FcγRII crystal complex) | KC101A | -0,03 | 1 | 0,00 |
| Double-free template model IgG1/FcγRIII (based on IgG1/FcγRII crystal complex) | EC102A | 0,19 | 1 | 0,00 |
| Double-free template model IgG1/FcγRIII (based on IgG1/FcγRII crystal complex) | EC103A | -0,07 | 1 | 0,00 |
| Double-free template model IgG1/FcγRIII (based on IgG1/FcγRII crystal complex) | DC104A | 0,6 | 1 | 0,00 |
| Double-free template model IgG1/FcγRIII (based on IgG1/FcγRII crystal complex) | SC112A | 0,04 | 1 | 0,00 |
| Double-free template model IgG1/FcγRIII (based on IgG1/FcγRII crystal complex) | WC113F | 1,73 | 1 | 0,07 |
| Double-free template model IgG1/FcγRIII (based on IgG1/FcγRII crystal complex) | QC125A | 1,36 | 1 | 0,02 |
| Double-free template model IgG1/FcγRIII (based on IgG1/FcγRII crystal complex) | NC126A | 1,56 | 1 | 0,00 |
| Double-free template model IgG1/FcγRIII (based on IgG1/FcγRII crystal complex) | CC127A | 0,96 | 1 | -0,03 |
| Double-free template model IgG1/FcγRIII (based on IgG1/FcγRII crystal complex) | KC128A | 1,02 | 1 | 0,05 |
| Double-free template model IgG1/FcγRIII (based on IgG1/FcγRII crystal complex) | DC129A | -0,46 | 1 | -0,81 |
| Double-free template model IgG1/FcγRIII (based on IgG1/FcγRII crystal complex) | RC130A | 1,85 | 1 | 0,85 |
| Double-free template model IgG1/FcγRIII (based on IgG1/FcγRII crystal complex) | RC130T | 1,48 | 1 | 0,90 |
| Double-free template model IgG1/FcγRIII (based on IgG1/FcγRII crystal complex) | KC131T | 0,76 | 1 | -0,79 |
| Double-free template model IgG1/FcγRIII (based on IgG1/FcγRII crystal complex) | YC132A | 1,56 | 1 | 0,31 |
| Double-free template model IgG1/FcγRIII (based on IgG1/FcγRII crystal complex) | AC144S | -0,27 | 1 | 0,00 |
| Double-free template model IgG1/FcγRIII (based on IgG1/FcγRII crystal complex) | TC145A | -0,34 | 1 | 0,00 |
| Double-free template model IgG1/FcγRIII (based on IgG1/FcγRII crystal complex) | SC149A | -0,39 | 1 | 0,00 |
| Double-free template model IgG1/FcγRIII (based on IgG1/FcγRII crystal complex) | CC150A | 1,36 | 1 | 0,00 |
| Double-free template model IgG1/FcγRIII (based on IgG1/FcγRII crystal complex) | SC151A | -0,08 | 1 | 0,00 |
| Double-free template model IgG1/FcγRIII (based on IgG1/FcγRII crystal complex) | SC160A | -0,2 | 1 | -0,07 |
